# Synaptic proteins in neuron-derived extracellular vesicles as biomarkers for Alzheimer’s disease: novel methodology and clinical proof of concept

**DOI:** 10.20517/evcna.2023.13

**Published:** 2023-03-31

**Authors:** Erez Eitan, Tricia Thornton-Wells, Katya Elgart, Eren Erden, Eve Gershun, Amir Levine, Olga Volpert, Mitra Azadeh, Daniel G. Smith, Dimitrios Kapogiannis

**Affiliations:** 1 NeuroDex Inc., Natick, MA 01760, USA.; 2 Alkermes, Inc., Department of Translational Medicine and Early-Stage Clinical Development, Waltham, MA 02451-1420, USA.; 3 National Institute on Aging (NIA/NIH), Human Neuroscience Section, Intramural Research Program, Baltimore, MD 21224, USA.; 4 Columbia University, Division of Child and Adolescent Psychiatry, Department of Psychiatry, College of Physicians and Surgeons, New York, NY 10032, USA.

**Keywords:** Biomarkers, exosomes, neuron-derived exosomes, Alzheimer’s disease

## Abstract

**Aims:**

Blood biomarkers can improve drug development for Alzheimer’s disease (AD) and its treatment. Neuron-derived extracellular vesicles (NDEVs) in plasma offer a minimally invasive platform for developing novel biomarkers that may be used to monitor the diverse pathogenic processes involved in AD. However, NDEVs comprise only a minor fraction of circulating extracellular vesicles (EVs). Most published studies have leveraged the L1 cell adhesion molecule (L1CAM) for NDEV immunocapture. We aimed to develop and optimize an alternative, highly specific immunoaffinity method to enrich blood NDEVs for biomarker development.

**Methods:**

After screening multiple neuronal antigens, we achieved NDEV capture with high affinity and specificity using antibodies against Growth-Associated Protein (GAP) 43 and Neuroligin 3 (NLGN3). The EV identity of the captured material was confirmed by electron microscopy, western blotting, and proteomics. The specificity for neuronal origin was demonstrated by showing enrichment for neuronal markers (proteins, mRNA) and recovery of spiked neuronal EVs. We performed NDEV isolation retrospectively from plasma samples from two cohorts of early AD patients (N = 19 and N = 40) and controls (N = 20 and N = 19) and measured p181-Tau, amyloid-beta (Aβ) 42, brain-derived neurotrophic factor (BDNF), precursor brain-derived neurotrophic factor (proBDNF), glutamate receptor 2 (GluR2), postsynaptic density protein (PSD) 95, GAP43, and syntaxin-1.

**Results:**

p181-Tau, Aβ42, and NRGN were elevated in AD samples, whereas proBDNF, GluR2, PSD95, GAP43, and Syntaxin-1 were reduced. Differences for p181-Tau, proBDNF, and GluR2 survived multiple-comparison correction and were correlated with cognitive scores. A model incorporating biomarkers correctly classified 94.7% of AD participants and 61.5% of control participants. The observed differences in NDEVs-associated biomarkers are consistent with previous findings.

**Conclusion:**

NDEV isolation by GAP43 and NLGN3 immunocapture offers a robust novel platform for biomarker development in AD, suitable for large-scale validation.

## INTRODUCTION

Developing effective treatments for Alzheimer’s disease (AD) and AD-related dementias (ADRD) represents an unmet medical need of major socioeconomic importance. Approved symptomatic treatments have no impact on disease progression, whereas therapeutic development remains exceptionally challenging and costly^[1]^. Only recently have two purportedly disease-modifying drugs, Lecanemab and Aducanumab, received FDA approval for the treatment of AD^[2]^; however, the clinical significance of their effects is questionable.

Complicating things further, postmortem analyses have shown that most dementia patients present with mixed underlying pathologies^[3–5]^. Therefore, as with cancer, a precision strategy informed by the patient’s underlying biology is highly desirable but currently underdeveloped. Detection of AD-specific pathologies in living patients currently relies on expensive and/or invasive biomarkers obtained through intensive positron-emission tomography (PET) scans or lumber punctures and the analysis of biomarkers in cerebrospinal fluid (CSF)^[6]^. And in both cases, these approaches measure and reflect only a limited number of pathologies focused on amyloid-beta (Aβ) and tau proteins. Blood biomarkers are inherently advantageous because blood draws are minimally invasive and can be performed repeatedly for early-stage diagnosis, monitoring of disease progression, and assessment of therapeutic responses^[7]^. A key challenge in developing blood biomarkers for neurodegenerative diseases is to achieve specificity for changes occurring in neurons and other brain cells rather than non-neuronal sources. One way to address this challenge is the enrichment of EVs isolated from blood plasma or serum for neuronal origin in order to analyze NDEVs. Because EVs are nanosized particles surrounded by a lipid bilayer membrane that contain proteins and RNA that are representative of their cells of origin^[8–12]^, they may provide a molecular snapshot of the brain^[9]^.

The use of NDEVs as a biomarker platform relies on a few assumptions backed with experimental support: the first is that the ability of NDEVs to cross the blood-brain barrier (BBB) has been demonstrated^[13–15]^; second, only NDEVs contain neuron-specific surface proteins^[16]^; and third, NDE cargo reflects the cell of NDE origin; and lastly, modifications to NDEV cargo are minimal following the release of NDEVs. Previous studies on biomarkers in EVs used antibodies against L1CAM (L1 cell adhesion molecule, CD171), a surface marker predominantly expressed by neurons but also by cells in the kidney, dermis, and peripheral lymphocytes (https://www.proteinatlas.org/ENSG00000198910-L1CAM/tissue), as a means to isolate NDEVs. L1CAM expression levels are relatively low in non-neuronal cells, but its presence in other tissues raises concerns regarding NDE origin and purity.

In the present study, we selected a combination of two from multiple antibodies against neuronal-specific markers that target two neuron-specific antigens: an axonal marker, growth-associated protein 43 (GAP43), and a neuron cell surface marker, neuroligin 3 (NLGN3)^[17,18]^. Our results indicate that the resulting isolation procedure adheres to MISEV guidelines^[19]^ and demonstrates its efficiency in recovery experiments where EVs released by iPSC-derived neurons are spiked into plasma samples. The specificity toward neuronal material is confirmed by measurements of multiple neuron-specific proteins and mRNA. We evaluated the performance of this technology for the assessment, detection, and quantification of biomarkers for AD in NDEVs, including p181-Tau, Aβ, pro-BDNF, and synaptic proteins, retrospectively, in samples from two cohorts of patients with early-stage AD and controls. These exploratory findings demonstrate the robust performance of the method and the potential to add novel, non-invasive, and scalable biomarkers to inform AD drug development, which deepens insight into neuronal pathology in AD.

## METHODS

### Standard protocol approvals, registrations, and consents

This study relied on de-identified samples from commercial or Government [National Institute of Aging (NIA, Baltimore, MD)] biobanks. Commercial samples were purchased from two companies, BioIVT (Westbury, NY) and precision for medicine (PMED) (Elk Grove, CA), which adhere to the most current regulations for sample collection and use. The regulatory requirements met include Institutional Review Board (IRB) approval, privacy officer authorization, appropriate government licenses, and industry accreditations, as applicable. They also adhere to the General Data Protection Regulations. Samples from the NIA were collected through NIH IRB-approved protocols, and their use in this research is allowed based on the subjects’ original consent. Regarding patients with AD, legally authorized representatives or patients consented to participate in the original studies, depending on the assessment of consent capacity at the time of blood draws. All samples were de-identified prior to their transfer to NeuroDex for processing under the terms of a Collaborative Research and Development Agreement.

#### Human plasma specimens:

Human EDTA-K2 plasma was used in all experiments. Pooled human EDTA-K2 plasma (BioIVT, HMN69947-X, ten donors per pool) was used for quality control experiments and as internal references. Samples procured from BioIVT and PMED comprised a single cohort for most analyses. The NIA cohort comprised 20 individuals with high probability (early-stage) AD as established by the NIA-AA criteria^[20]^ and 19 healthy, cognitively normal age- and sex-matched controls, participating in NIH IRB-approved studies (NIA Clinical Unit; Baltimore, MD, USA). AD diagnosis was based on clinical criteria and abnormal CSF levels of amyloid-beta (Aβ) peptide 1–42 (Aβ42, < 192 pg/mL) and p181-Tau > 23 pg/mL^[21]^. Demographic and clinical data for all cohorts are summarized in Table 1. All NeuroDex employees involved in sample processing and analysis were blinded to the identity of the samples, and only internal sample IDs were used.

#### NDEV isolation:

NDEV enrichment was performed using the NeuroDex ExoSORT kit (Cat. No NDX_ESNeuro, NeuroDex, Natick, MA). Briefly, plasma samples were precipitated with 1/2 plasma volume of a NeuroDex total EV isolation reagent (Cat. No. NDX_TPC) in 1/2 plasma volume, and pellets were resuspended in a binding buffer. Magnetic beads were conjugated with NeuroDex proprietary antibodies against GAP43 and NLGN3 and blocked with the NDX Blocking Reagent. The beads were incubated overnight with plasma samples at 4 °C with slow rotation. The following day, beads-NDEVs complexes were collected using a magnetic separator, washed three times using ExoSORT wash buffer, and transferred into ExoSORT elution buffer. The elution of EVs was performed for 5 min at 50 °C, followed by the removal of the beads. Eluates were collected and transferred into clean tubes with ExoSORT lysis buffer.

#### RNA isolation:

NDEVs captured on ExoSORT beads, as described above, were washed twice with NDX ExoSORT wash buffer (provided in the kit) and transferred to QIAzol reagent (Qiagen, Cat. No. 79306). RNA was isolated using the miRNAeasy serum and plasma kit (Qiagen, Cat. No. 740004) according to the manufacturer’s instructions.

#### PCR analysis:

cDNA was generated using SuperScript^™^ IV VILO^™^ reagent mix (Thermo-Fisher, Cat. No. 11766050). qPCR was performed using TaqMan master mix (Thermo Fisher Scientific, Cat. No. 4305719) and predesigned TaqMan gene expression assays (see Table 2) in a Step One Thermal Cycler (Applied Biosystems, Cat. No. 4369074). The data were expressed as ΔCt, without using any gene for normalization, as there is no consensus in the field on a normalization gene for plasma EV RNA.

#### EV isolation from cell culture media:

Conditioned media (CM) from a culture of cortical neurons differentiated from induced pluripotent stem cells (iPSCs) and maintained for 4–8 weeks in differentiation media) was obtained from BrainXell (Cat. No. BX-0200). The cells were maintained in a proprietary serum-free, EV-free medium. CM was centrifugated at 1,000 × g for 10 min, and supernatants were collected and centrifugated at 3,000 × g for an additional 10 min. Multiple collections were combined for a total of 200 mL, and EVs were isolated by ion exchange chromatography (IEC) and concentrated by ultrafiltration as previously described^[22]^. Briefly, CM was loaded on Q Sepharose Fast Flow columns (Cytiva, Cat. No. 17–0510-10). The unbound material was washed off with equilibration buffer, followed by wash and elution with buffers containing sequentially increasing concentrations of NaCl. The EVs were concentrated by dialysis in 100 kDa MWCO spin filtration units (Pall Corporation, Cat. No. MAP100C38) against NeuroDex storage buffer supplemented with protease and phosphatase inhibitors (Thermo Fisher Scientific, Cat. No. 78429, 78426).

#### Immunoassays:

Tau, p181-Tau, Aβ40, and Aβ42 were measured according to the manufacturer’s instructions in undiluted NDEVs lysates using commercial Luminex kits (EMD Millipore, Cat. No. MXHABTM0N02010 and HNABTMAG-68K-04). Mature BDNF and proBDNF were measured in NDEVs lysates diluted 1:10 using a commercial Enzyme-Linked Immunosorbent Assay (ELISA) kit (Biosensis, Cat. No. BEK-2241). Apolipoprotein A (ApoA) (R&D system DY3664–05) and Albumin (R&D system DY1455) were also measured using commercial ELISA kits according to manufacturers’ instructions. Rab3a, GLUR2, NLGN3, and L1CAM, as well as the EV markers Flotillin (FLOT)-1 and CD9, were measured by intact EV ELISA as described elsewhere^[23]^. FLOT-1 and CD9 values were used to normalize ELISA results since the high content of non-EV particles in plasma challenges the accuracy of particle counts by NTA or light scatter.

Intact EV Luminex analysis was performed with the NeuroDex Lumin-EV kit (NDX_LUMTET). Briefly, MagPlex microspheres (MC100XX-01) were conjugated with antibodies against CD9, CD63, CD81, or with antibodies against synaptic proteins using an ABC coupling kit (Luminex Corp., Cat. No. 4050016). The resultant capture beads were used in a multiplex format to capture EVs directly from plasma as described previously^[22]^. The captured EVs were detected with a pan-tetraspanin antibody cocktail (for specific antibodies, see Table 3).

An intact EV ELISA assay was performed with the NeuroDex ELISA kit (NDX_ELISA). High-binding plates (Corning, Cat. No. 9018) were coated overnight with antibodies against the synaptic proteins of interest (antibody catalog numbers in Table 3). Then, the plates were blocked for 2 h, washed, and incubated for 2 h with detection antibodies at room temperature on a plate shaker. Next, the plates were washed three times, and biotinylated GAP43 antibody was added for 2 h, followed by a wash step. Next, streptavidin-HRP was added for 30 min, and the plates were washed and developed by TMB.

#### Internal reference/standards:

For all assays, two internal reference standards were included, comprising pre-evaluated pooled human plasma or NDEV isolation, as appropriate. In cases of inconclusive or missing reference data, the test was repeated.

#### Transmission electron microscopy:

Isolated NDEVs resuspended in 3% PFA and incubated with negative stain (Uranyl Acetate) were visualized at the Brandeis University Cell Imaging Facility (Dr. Berith Isaaks) using a Morgagni transmission electron microscope (FEI, Hillsboro, OR), operating at 80 kV and equipped with a Nanosprint5 CMOS camera (AMT, Woburn, MA).

#### Western blotting:

NDEVs protein lysates and reference samples were processed using the WES apparatus and 25-sample cartridges containing loading buffer and secondary antibodies (ProteinSimple), according to the manufacturer’s instructions (for specific antibodies, see Table 3).

#### Proteomic analysis:

Isolated NDEVs samples were sent to Tymora Analytical LLC for proteomic analysis using a proprietary procedure optimized for EV analysis.

#### Lipidomic analysis:

Lipidomic profiling of NDEVs was performed by ultra performance liquid chromatography-tandem mass spectrometry (UPLC-MSMS) at the Columbia University Lipidomic Facility, as described previously^[24]^.

#### Nanoparticle tracking analysis:

NDEV preparations were diluted 10 times in pre-filtered PBS (20 mm filters), and NTA analysis was performed using NanoSight 500 (Malvern Panalytical) as described previously^[25]^.

### Statistical analysis

Statistical analysis was conducted with SPSS v27.0 (IBM). To assess group differences for individual biomarkers, we used linear mixed models including each biomarker as an independent variable, sex and group (early-stage AD *vs*. control) as factors, and age as a covariate. Pairwise comparisons were assessed using least-squares means. Although this exploratory study was not powered to correct for multiple comparisons, when results met Bonferroni correction (i.e., significance level *P* < 0.003125 for 8 independently measured biomarkers in 2 independent cohorts), this is noted in Results and Figures. To assess whether results may be replicable across diverse populations, the NIA and BioIVT/PMED Cohorts were analyzed separately. We also assessed performance in diagnostic classification for AD/MCI *vs*. control status. To determine the simplest and most accurate classifier model based on multiple biomarkers, we performed discriminant classifier analysis stepwise with the Wilks’ Lambda method, allowing biomarkers to “compete” against each other in each step with a minimum partial F of 3.84 to enter and 2.71 to remove. To determine the ability of individual biomarkers in group classification, receiver operator characteristic (ROC) analysis was conducted under the non-parametric distribution assumption. To assess the relationships among biomarkers and between biomarkers and clinical and cognitive scores, we computed zero-order and partial Pearson correlations (controlling for age and sex). Discriminant, ROC, and correlation analyses were conducted after combining all cohorts into one.

#### Data availability:

De-identified data are available upon request from a qualified investigator.

## RESULTS

### ExoSORT captures extracellular vesicles

**Figure 1.**
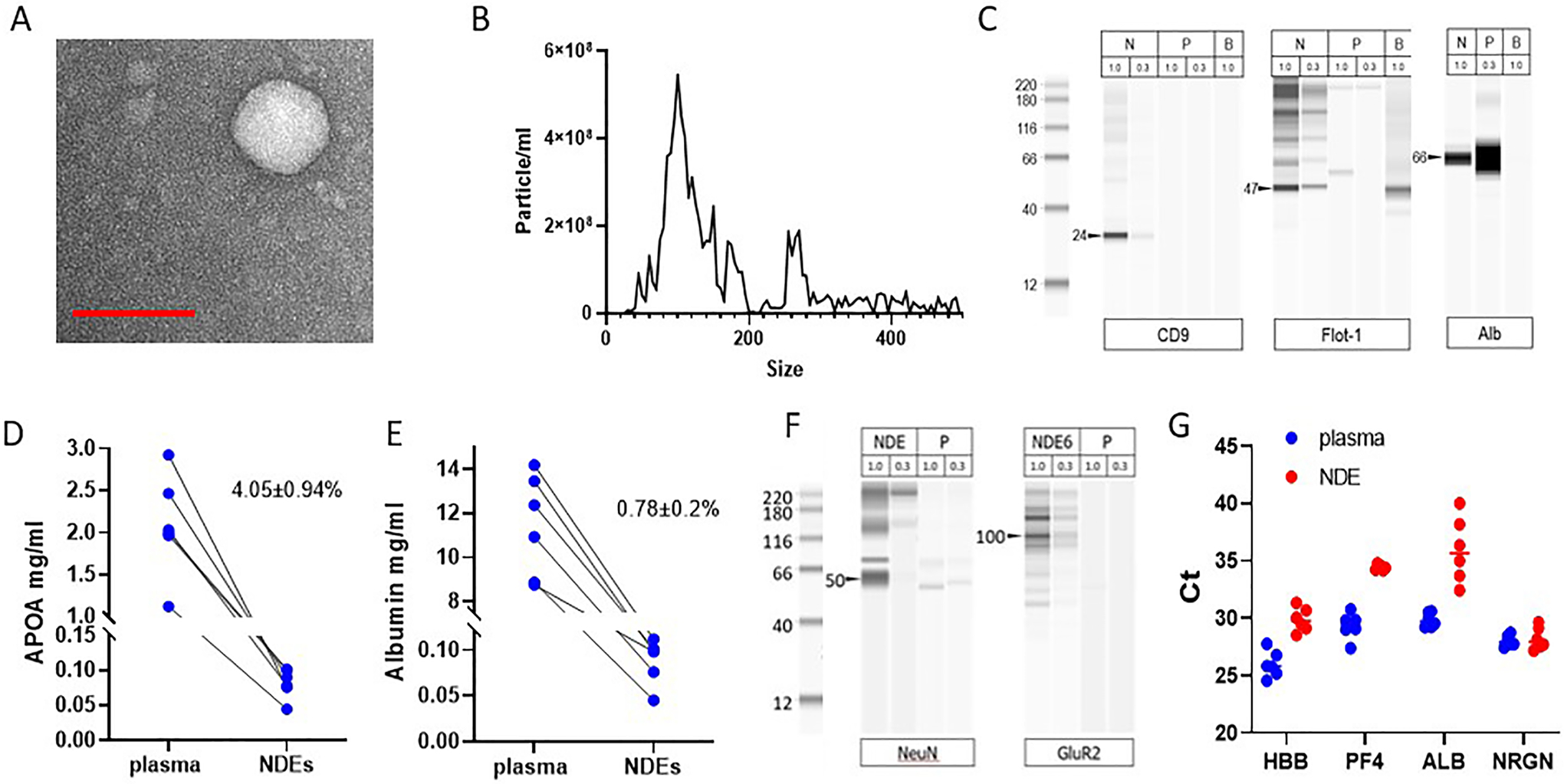
NDEV isolation and characterization. The isolated NDEVs demonstrate key EV characteristics. (A) NDEVs were eluted from ExoSORT capture beads, fixed in 3% PFA, and visualized by transmission electron microscopy. Vesicular shape and size under 200 nm were observed. (B) The size distribution of eluted NDEVs was determined by nanoparticle tracking analysis. The NDEVs average diameter ± SEM was 127 ± 72 nm. (C) Western blot analysis (WES instrument) showed that NDEVs (N) contained enriched levels of typical EVs markers CD9 and FLOT1, and reduced levels of albumin, compared to unprocessed plasma (P). Mouse brain extract (B) was used as a control. Total protein loaded (ug) is shown for each lane. (D, E) Levels of albumin and ApoA1 were much lower in isolated NDEVs than in unprocessed plasma, as quantified by ELISA (R&D Systems, Cat. No. DY1455 and DY366405). The fractions of albumin and ApoA1 in NDEVs were 0.78 ± 0.2% and 4.05 ± 0.94% of the levels observed in unprocessed plasma, respectively (*P* < 0.0001 for both proteins). (F, G) The enrichment for neuronal material was ascertained by comparing neuron-specific proteins and RNA in plasma and NDEVs. (F) WES analysis showed that NDEVs (lanes labeled N) are enriched for neuronal markers NeuN and GluR2 compared to unprocessed plasma (lanes labeled P). Protein amounts loaded onto each lane are shown above. (G) mRNA encoding markers of EV origin from erythrocytes (hemoglobin, HBB), platelets (platelet factor 4, PF4), liver (albumin, ALB), and neurons (neurogranin, NRGN) were measured by QPCR in plasma and NDEVs. The resultant values were adjusted to the input volume. Note that there were lower levels of HBB, PF4, and ALB mRNA in NDEVs compared to plasma (1,4, 34, and 68-fold, respectively; *P* < 0.0001 for all comparisons). In contrast, NRGN mRNA levels were similar between NDEVs and plasma (*P* = 0.66).

ExoSORT is a novel immunoaffinity-based method for EV isolation and was therefore evaluated for compliance with MISEV guidelines^[19]^ [Figure 1]. Spherical morphology was shown by electron microscopy [Figure 1A], and average particle diameter in the 50–250 nm range was confirmed by nano-tracking analysis (NTA; Figure 1B), even though it seems that NDEVs display a non-normal size distribution. The presence of typical EV markers, CD9 and flotillin (FLOT1), as well as reduced levels of the non-EV protein albumin, were shown by Western blot/WES [Figure 1C]. The depletion of contaminating plasma proteins was assessed using ELISAs for Apolipoprotein A1 (ApoA1) and albumin, with depletion of 95.95 ± 0.94% and 99.8 ± 0.2%, respectively (N = 6, *P* < 0.001; Figure 1D and E). The relatively higher level of ApoA1 contamination compared to albumin is consistent with previous reports demonstrating EV interactions with HDL/LDL^[26]^. Lipidomic analysis showed high concentrations of characteristic EV lipids, such as cholesterol, phosphatidylserine, and sphingomyelin [Supplementary Figure 1A and Supplementary Table 1A], and unbiased proteomic analysis revealed multiple EV-specific proteins in the NDEV preparations [Supplementary Figure 1A and Supplementary Table 1B].

### NDEVs captured by ExoSORT are enriched for neuronal markers

NDEVs captured by ExoSORT showed higher levels of neuron-specific proteins and 24-hydroxycholesterol, a lipid enriched in the brain^[27]^, compared to the procedural control, material immunocaptured using IgG isotype antibody [Table 4]. The enrichment of neuronal proteins was also demonstrated by WES with antibodies against neuron-specific proteins, GluR2 and NeuN [Figure 1F]. Unbiased proteomic analysis also revealed significant enrichment for neuronal proteins, as was shown by gene ontology analysis [Supplementary Table 2]. Since EVs are the major carriers of circulating mRNAs^[28]^ and RNA measurements are less sensitive to matrix effects, neuron-specific mRNA content was compared between NDEVs and unprocessed plasma [Table 4 and Figure 1G]. The NDEV levels of mRNA encoding for hemoglobin beta chain (HBB, erythrocyte marker), as well as platelet factor 4 (PF4, platelet marker), and albumin (ALB, liver marker), were less than 6% of those in unprocessed plasma, suggesting successful depletion of non-specific material; in contrast, the levels of mRNA encoding for the neuronal marker NRGN were similar in NDEVs and plasma, pointing to efficient recovery of neuron-specific EVs [Figure 1G]. The levels of neuronal markers in NDEVs were 35 times higher than in the material isolated with control IgG (procedural control, *P* < 0.0001; Table 4). The diminished levels of non-neuronal mRNAs and the preservation of neuronal mRNAs and proteins corroborate the specificity of the NDEV isolation.

### ExoSORT isolates endogenous and spiked NDEVs with high efficiency and precision

**Figure 2.**
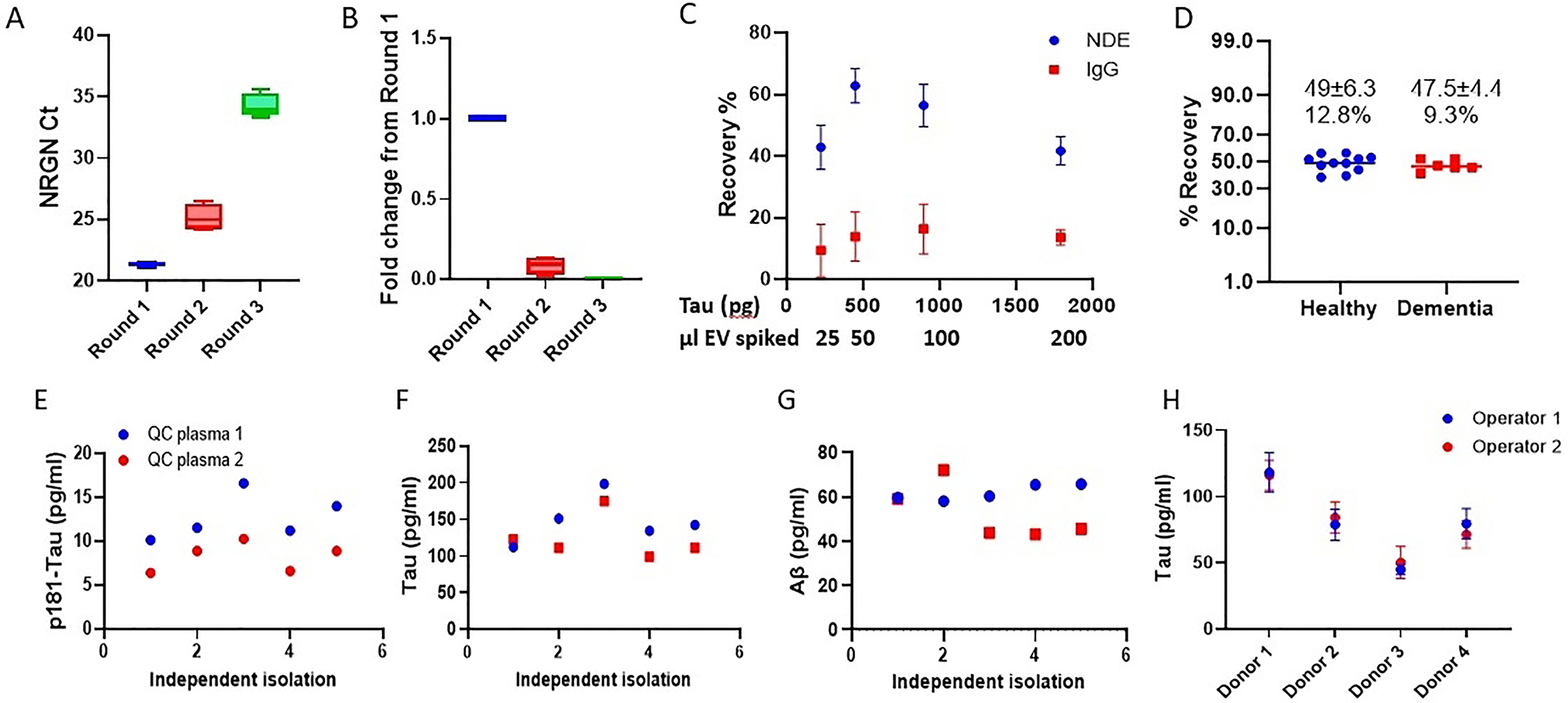
Efficiency and precision of NDEV isolation. (A) NDEV isolation was performed on unprocessed plasma samples (Round 1, N = 4, aliquoted from the same plasma pool). After the first round of ExoSORT, supernatants were retained and subjected to an additional round of capture with fresh ExoSORT beads (Rounds 2 and 3, see Supplementary Figure 3). NRGN mRNA was measured by TaqMan QPCR. Ct values are shown. (B) Tau-rich EVs generated by iPSC-derived neurons were spiked into plasma aliquots. The recovery of spiked NDEVs in three consecutive rounds of ExoSORT was measured as above, and residual NDEV amounts after each round were estimated. Fold change differences between initial plasma pools (Round 1) and the two consecutive rounds are shown (Rounds 2 and 3). The signals in Rounds 2 and 3 were 8.7 ± 5.5% and 0.02 ± 0.01% in Round 1, respectively. (C) EVs isolated from a culture of human iPSC-differentiated cortical neurons were spiked into 300 ml plasma aliquots in 4 different concentrations based on known amounts of Tau. EV recovery was calculated by the following formula: $\text{Recovery}=\frac{\text{Tau concentration in spiked sample}-\text{Tau concentration in non–spiked sample}}{\text{Tau input}}$. Tau recovery after ExoSORT remained uniform for a range of spiked-in EV-associated Tau amounts and was much higher than with IgG control (*P* = 0.026, two-way ANOVA). (D) Identical EV amounts from a culture of human iPSC-differentiated cortical neurons were spiked into plasma samples from 11 healthy individuals and six dementia patients. The variability of recovery was < 13% for all samples, with no difference between groups (*P* = 0.6). (E-G) Five different ExoSORT procedures were performed on different days, using aliquots derived from the same two plasma samples (QC1 and QC2, respectively). The levels of p181-Tau, total-tau, and Aβ42 were measured in the same assay (Luminex-based Milliplex), yielding variability estimates of 20.3%, 22.7%, and 14.9% for p181-Tau, Tau, and Aβ42, respectively. (H) ExoSORT was performed in triplicate technical replicates by two operators on plasma from 4 distinct donors, and the results were compared using Tau levels as an output. We note similar variations between donors and similar levels determined by different operators for each donor (*P* = 0.83).

To estimate the NDEV isolation efficiency, we compared NRGN mRNA levels in NDEVs isolated by ExoSORT and in unprocessed plasma and found that NRGN levels in NDEVs comprised 86 ± 7% of those in the parent plasma [Figure 1G]. Further estimates of NDEV isolation efficiency were based on conducting sequential rounds of ExoSORT, wherein the supernatant remaining after capture bead removal in each round was retained and subjected to a new round of ExoSORT [Supplementary Figure 2]. With NRGN mRNA level used as a measure of NDEVs extraction, the difference between two sequential yields was 87 ± 9% providing a measure of isolation efficiency [Figure 2A and B]. Next, we used a classical spiking approach using EVs isolated from human iPSC-derived cortical neurons. Culture-derived EVs, which were characterized according to MISEV guidelines^[19]^ [Supplementary Figure 3A–C], contain exceptionally high Tau levels [Supplementary Figure 3D], at least ten times higher than those expressed in endogenous plasma NDEVs. This difference enabled the use of Tau measurements to estimate spiked iPSC EV recovery from plasma since their contribution far exceeds that of endogenous NDEVs. iPSC EV in amounts containing 250–2000 pg Tau (30–200 ul) was spiked into 300 ml plasma aliquots, and Tau recovery was measured using a Luminex-based Milliplex assay (EMD Millipore). The recovery of the spiked reference material was estimated at 51 ± 10%, and the efficiency was not dependent on the input amount (N = 4, Figure 2C). The same spiking approach was used to evaluate the consistency between NDEVs recovery from plasma samples of disease-free controls (N = 11) and AD patients (N = 6) [Figure 2D]. These results further demonstrate the compatibility of the NDEV isolation methodology with the plasma matrices of both control and AD individuals. Of note, each of the capture antigens used in ExoSORT offered an improvement in NDEV capture compared to L1CAM [Supplementary Figure 4].

ExoSORT precision was also evaluated in the absence of spiked reference standards, yielding a coefficient of variance (CV) between 8.0 and 22.7% for two random plasma samples processed repeatedly in five independent experiments [Figure 2E–G]. The variability between two proficient operators was lower than the variability between donors (N = 4, CV < 21%; Figure 2H). Moreover, the isolation of NDEVs was successfully performed by a proficient operator in a different lab (the Kapogiannis lab at the NIA) [Supplementary Figure 5]. Importantly, the estimated variability combines the variance from two sources: NDEV isolation (ExoSORT) and biomarker measurements (ELISA or Luminex).

### NDEVs-associated p181-Tau and Aβ42 confirm ExoSORT diagnostic potential and robust performance

**Figure 3.**
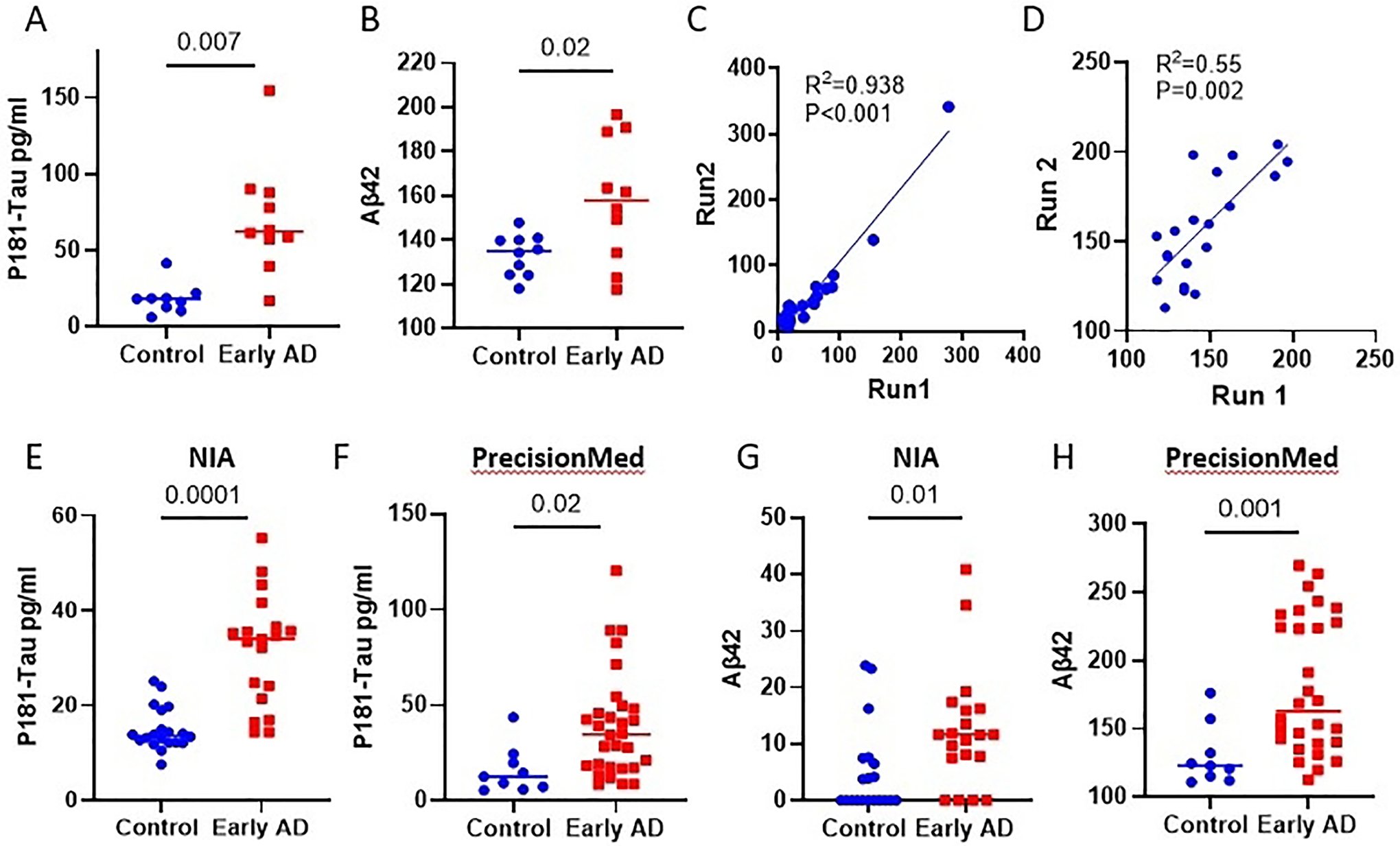
p181-Tau and Aβ42 in NDEVs in three cohorts. The levels of p181-Tau, total Tau, and Aβ42 were measured in three cohorts of early-stage AD patients and control donors. (A, B) NDEVs-associated p181-Tau and Aβ42 were measured by a Milliplex assay in the BioIVT cohort (N = 10 per group, *P* = 0.007 and *P* < 0.0001, respectively). (C, D) Different aliquots of the same 20 plasma samples from the BioIVT cohort were analyzed again at a later date to assess the correlation between the two measurements (R^2^ = 0.93, *P* < 0.01 and R^2^ = 0.55, *P* < 0.002, respectively). (E, F) p181-Tau measurements were conducted in NDEVs derived from NIA samples (20 early AD and 19 controls, *P* < 0.0001) and PMED samples (30 early AD and 9 controls, *P* = 0.02). (G, H) NDEVs-associated Aβ42 was measured by Luminex in the NIA and PMED cohorts, and significant differences were detected between the disease and control groups (*P* = 0.01 and *P* = 0.001, respectively).

The diagnostic potential of the NDEVs isolated using L1CAM-based immunocapture has been demonstrated by multiple groups, including the authors of this study^[29,30]^. To examine the ability of ExoSORT-derived NDEVs to also detect previously reported biomarker differences, we first tested their performance in a cohort of 10 early-stage AD patients and 10 age-matched control participants (BioIVT). Similar to reports using L1CAM isolation, p181-tau and Aβ42 levels were higher in AD samples compared to control specimens [Figure 3A and B]. The robust assay performance was ascertained by repeated analyses of independently stored aliquots of the same samples [Figure 3C and D], yielding a strong correlation for p181-Tau (R^2^ = 0.93 *P* < 0.001) and a medium-strength correlation for Aβ42 (R^2^ = 0.55, *P* = 0.002). Furthermore, similar results were obtained in two additional well-characterized cohorts (20 high-probability early AD samples and 19 control samples from the NIA and 30 early AD samples, and nine control samples from PMED) [Figure 3E–H]. Importantly, the differences were statistically significant, and the direction of the differences between control and AD samples was the same across different cohorts; however, the absolute Aβ42 levels varied across cohorts, suggesting assay sensitivity to preanalytical conditions unique to each cohort.

### NDEVs-associated proBDNF shows potential as a biomarker for AD

**Figure 4.**
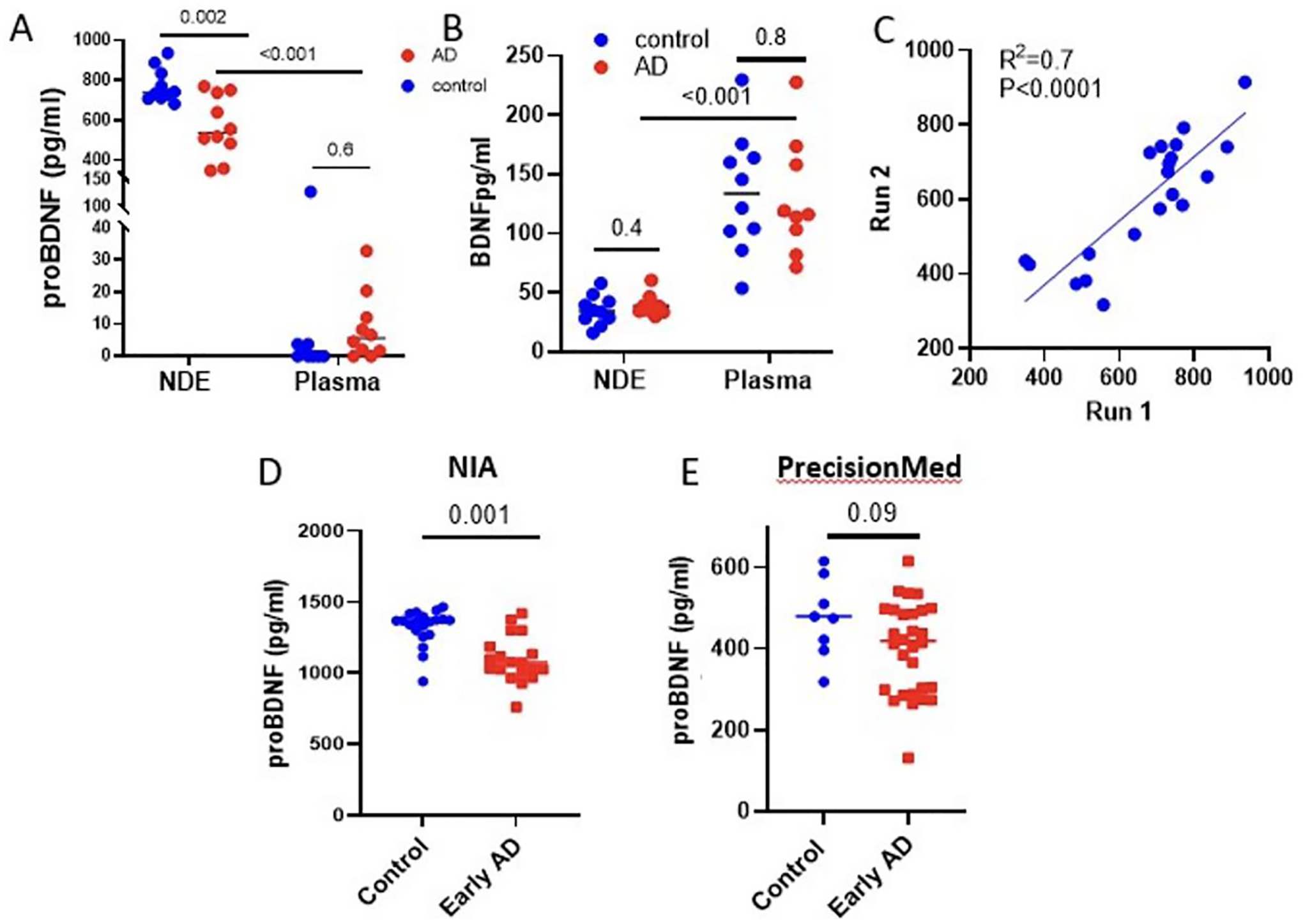
NDEVs and their source plasma samples were used to measure BDNF and proBDNF in early AD and control donors. (A) Measurements in the BioIVT cohort showed high enrichment of proBDNF levels in NDEVs (*P* < 0.001) compared to unprocessed plasma. Moreover, we observed lower levels of NDEVs-associated proBDNF in early AD compared to control individuals (*P* = 0.002), while no difference was observed in unprocessed plasma. (B) BDNF was 2.6-fold lower in NDEVs than in plasma (*P* < 0.01) and did not vary between AD and controls. (C) ProBDNF measurements in two independently stored aliquots from 20 samples showed a moderately strong correlation (R^2^ =0.7, *P* < 0.0001). (D, E) NDEVs-associated proBDNF was measured in two additional cohorts from NIA and PMED; the decrease in NDEVs-associated proBDNF in early AD compared to control individuals was reproduced in the NIA cohort (*P* = 0.001), while the PMED cohort generated a similar trend that did not reach statistical significance (*P* = 0.09), potentially due to an insufficient number of control samples.

Using ExoSORT, we reproduced published findings, wherein proBDNF, rather than mature BDNF, was predominantly found in association with NDEVs^[31]^ [Figure 4A and B]. This also provides further support for the enrichment of NDEVs for neuronal cargo, as their levels of proBDNF were much higher than in plasma. Interestingly, only NDEVs-associated proBDNF was significantly reduced in early AD compared to control samples [Figure 4A], while mature BDNF showed no differences [Figure 4B], suggesting yet another advantage of NDEV isolation for biomarker discovery. Assay reproducibility was verified by repeated analysis of multiple aliquots from the same sample (Figure 4C; R^2^ = 0.7, *P* < 0.001), and the significance and direction of the differences were reproducible between the NIA and PMED cohorts [Figure 4D and E]. However, similar to Aβ42, levels of proBDNF varied widely between cohorts, again suggesting sensitivity to preanalytical conditions.

A comparison with CSF levels of classical AD markers revealed a significant negative correlation between NDEVs and CSF levels of Aβ [Supplementary Figure 6], consistent with a similar finding by Jia *et al*.^[32]^.

### NDEV-associated synaptic proteins present biomarker potential for AD

**Figure 5.**
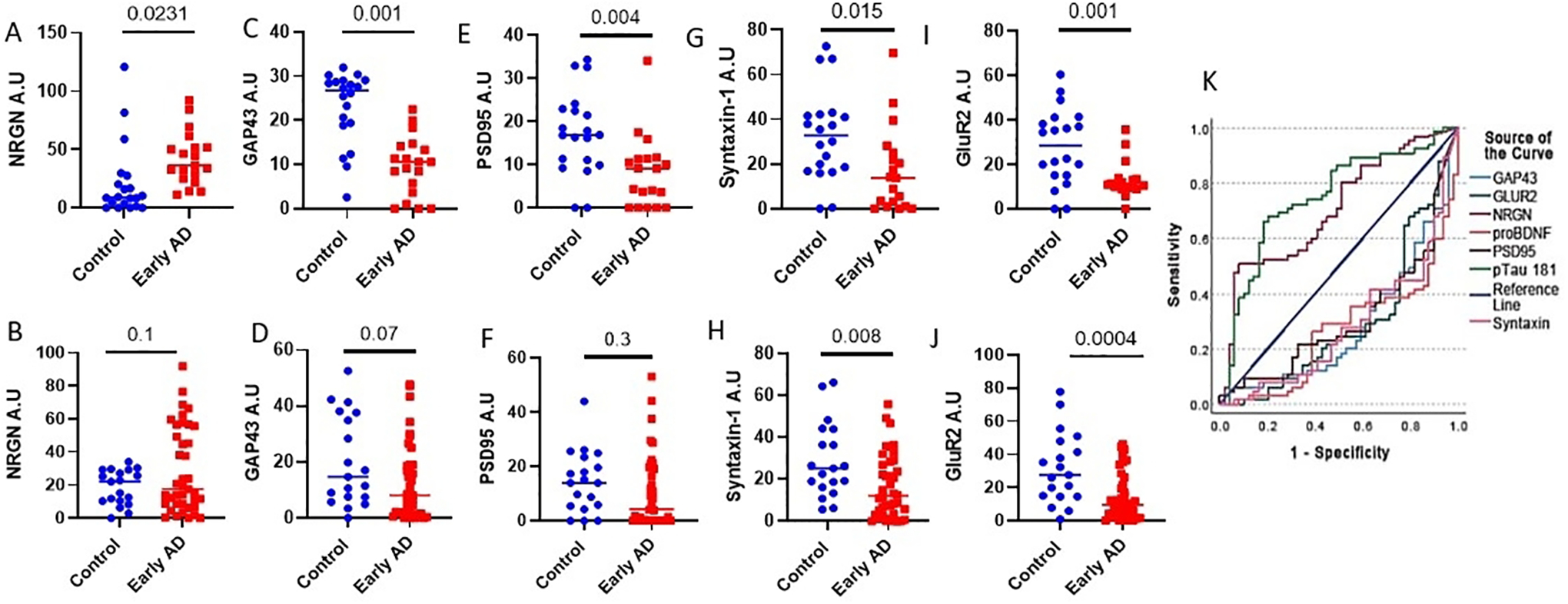
The levels of five synaptic proteins, NRGN, GAP43, PSD95, Syntaxin-1, and GluR2, were measured in three independent cohorts. (A-I) NDEVs-associated NRGN, GAP43, PSD95, Syntaxin-1, and GluR2 were measured in the NIA cohort (20 AD and 19 controls; NRGN: *P* = 0.025, GAP43: *P* < 0.001, PSD95: *P* = 002, Syntaxin-1: *P* = 0.011, and GluR2: *P* < 0.001). (B-J) The same proteins were measured in the combined BioIVT/PMED cohort (40 AD and 19 controls total), NRGN: *P* = 0.087, GAP43: *P* = 0.063, PSD95: *P* = 0.2, Syntaxin-1: *P* = 0.03 and GluR2: *P* < 0.001). (K) ROC analysis was conducted for all three cohorts combined. Each line corresponds to a different NDEV biomarker; higher values indicate AD/MCI, and lower values indicate CN Control status.

Synaptic loss is a major pathological feature of AD. Previous studies have shown high levels of synaptic proteins in NDEVs^[33–35]^. Many of these proteins were notably decreased in NDEVs of AD patients compared to control donors, and in some cases, these changes correlated with cognitive performance^[33]^. Here, we measured five NDEVs-associated synaptic proteins: Syntaxin 1 (STXN1), GAP43, GluR2, PSD95, and NRGN. In the NIA cohort, the levels of NDEVs-associated NRGN were elevated in AD samples compared to controls, whereas GAP43, PSD95, STXN1, and GluR2 were decreased (Figure 5A–E; NRGN, *P* = 0.025; GAP43, *P* < 0.001; PSD95, *P* = 0.002; STXN1, *P* = 0.011; GluR2, *P* < 0.001). The AD-associated decreases in NDEV-associated STXN1 and GluR2 were also present in the combined BioIVT-PMED cohort (*P* = 0.031 and *P* < 0.001, respectively). However, for NRGN, PSD95, and GAP43, directionally consistent trends did not reach significance, with *P* = 0.1, *P* = 0.07, and *P* = 0.3, respectively [Figure 5F–J]. Importantly, for GluR2 and proBDNF, the differences between early AD and controls were reproducible across multiple cohorts and, moreover, survived Bonferroni correction for multiple comparisons (i.e., *P* < 0.003125). The biomarkers examined in this study showed neither age- nor sex-associated differences.

### Performance of multiple NDEV biomarkers in AD diagnosis

ROC analysis for the classification of individual samples as belonging to early AD *vs*. control diagnostic categories [Figure 5K] showed that p181-Tau had the highest AUC (AUC = 0.832, *P* < 0.001), which was closely followed by GluR2 (AUC = 0.755, *P* < 0.001), proBDNF (AUC = 0.736, *P* < 0.001), GAP43 (AUC = 0.729, *P* < 0.001), NRGN (AUC = 0.716, *P* = 0.001), STXN1 (AUC = 0.709, *P* = 0.001), and PSD95 (AUC = 0.675, *P* = 0.005). Stepwise discriminant analysis for early AD *vs*. control classification incorporated stepwise GluR2, proBDNF, NRGN, and GAP43. The final model (cross-validated by leaving 1 out) yielded AD classification with 81.3% accuracy (*P* < 0.001), 94.7% sensitivity, and 61.5% specificity. Finally, we computed partial correlations (controlling for age and gender) in early-stage AD participants to examine the relationship between NDEV biomarkers and clinical scores. p181-Tau, proBDNF, and GluR2 were significantly associated with the MMSE and the Clinical Dementia Rating sum of boxes (CDR-SOB) [Table 5].

## DISCUSSION

The need for non-invasive, scalable, and reliable biomarkers to inform patient selection, monitor disease progression, and demonstrate target engagement is among the greatest challenges for successful drug development for neurodegenerative brain disorders. Blood collection for biomarker assessment is inherently less invasive than CSF sampling and more scalable than brain imaging, the two most common current approaches for the diagnosis and monitoring of neurodegenerative diseases, especially AD. EVs are increasingly recognized as a promising platform for biomarker discovery in neurological and psychiatric diseases^[36]^; however, technical difficulties hinder their clinical implementation^[37]^. The heterogeneity of plasma EVs due to differences in their biogenesis and cellular origins heightens the challenge but also provides opportunities for identifying multiple interesting and informative EV sub-populations using cell-origin-specific capture antigens^[38–41]^.

Here we demonstrate NDEV enrichment by selective immunocapture of EVs by two surface antigens, GAP43 or NLGN3, the expression of which is highly neuron-specific^[18,42]^. Although GAP43 is expressed predominantly in the brain, it can also be found in some cases of colorectal cancer and inflammatory bowel disease^[43]^. GAP43 expression in the gut, even in these conditions, is low in comparison to the brain. However, it is important to note that ExoSORT, like any immunoaffinity method, depends on the specificity of the selection marker, which is rarely completely specific. The data we presented demonstrate that we achieved significant enrichment of neuronal-specific cargo (proteins and RNA) through ExoSORT with GAP43 and NLGN3 antibodies.

Our method specificity for EV capture was corroborated by transmission electron microscopy, nanoparticle tracking analysis, western blotting, and ELISA measurement of EV-specific and non-EV (negative) markers^[26,44,45]^, as well as by unbiased proteomic and lipidomic analyses, according to the MISEV 2018 guidelines^[19]^. Interestingly, we found that contamination of NDEVs by ApoA was stronger than albumin, consistent with reports regarding EV and HDL/LDL interactions^[26]^. Measurement of multiple neuron-specific proteins and mRNAs^[27]^ showed dramatic enrichment in NDEVs isolated by ExoSORT compared to a procedural control (non-specific IgG), strongly supporting the specificity of ExoSORT towards neuronal EVs.

Since most blood mRNA species are enclosed in EVs^[28,46,47]^, we compared NDEVs and plasma levels of neuronal mRNAs and mRNAs originating from highly abundant blood components (erythrocytes and platelets) and the liver. While non-neuronal mRNA levels in NDEVs were reduced by nearly 20-fold compared to plasma, neuronal mRNA levels were similar between NDEVs and plasma, suggesting high capture specificity and efficiency. The high efficiency of ExoSORT was further confirmed by ascertaining high recovery and low residues in consecutive ExoSORT rounds (see Supplementary Figure 2). Importantly, NDEV recovery was consistent between experiments and plasma samples. Interestingly, the recovery of spiked EVs from cultured iPSC-derived cortical neurons seems to be lower than that of endogenous plasma NDEVs. It is possible that GAP43 and NLGN3 presentation differs between endogenous plasma NDEVs and iPSC-derived cortical neurons EVs. Differences between neuronal EVs present in the brain extracellular milieu and plasma NDEVs require further investigation, especially to understand whether specific subsets of brain EVs cross into the blood. The precision of the entire methodology was measured by analyzing pooled plasma on different days, with repeat analysis of clinical samples being below 20%. Isolation of NDEVs by ExoSORT was also reproducible between operators and labs. To the best of our knowledge, this is the first quantitative analysis of NDEV isolation efficiency, which is an integral step required by FDA regulatory guidelines for analytical validation^[48]^.

To assess the potential of NDEVs obtained using ExoSORT as a biomarker platform, NDEVs were isolated from retrospective cohorts of individuals with early AD and controls. These NDEVs were used to measure a variety of biomarkers, including the “classical” AD biomarkers, p181-Tau, and Aβ42. Importantly, both were significantly higher in AD samples compared to those of control individuals in multiple cohorts, in agreement with previous reports that relied on L1CAM-based NDEV capture^[10,29,49,50]^. Moreover, we observed a similar inverse correlation between NDEVs and CSF for the Aβ42/Tau ratio^[32]^.

Synaptic dysfunction and loss are early and persistent features of AD that, for some time, precede neuronal cell loss^[51]^. Several synaptic proteins in the CSF have also been suggested as AD biomarkers^[51–53]^. Complex roles of EVs in synaptic plasticity have been documented^[52]^, while multiple synaptic proteins have been detected in circulating L1CAM NDEVs^[34,35,54]^. Here, we measured synaptic proteins using a novel sandwich immunoassay for intact EVs with a common capture antibody (GAP43) and varying detection antibodies. NDEV-associated Syntaxin-1 and GluR2 were found to be lower in early AD compared to controls in multiple cohorts. NRGN PSD95 and GAP43 showed abnormal levels in the NIA cohort, although not in the combined BioIVT/PMED cohort. GluR2, proBDNF, and p181-Tau also showed significant correlations with MMSE and CDR-SOB. We should bear in mind that this study was not designed to examine associations with cognition across the entire range of AD severity since all participants had the early disease and their cognitive score range was narrow. It is also interesting that, unlike other synaptic proteins, NRGN was higher in early AD compared to controls. Whether this finding represents a specific biological process (such as ongoing synaptic damage) in AD remains to be determined by mechanistic studies.

Reproducibility between cohorts is of high importance for biomarker development^[55,56]^. Here, we used multiple cohorts to examine the robustness of NDEV isolation. While most differences between early AD and control groups were consistent across cohorts, the absolute levels of several biomarkers varied widely between cohorts. We consider it likely that these differences were partly due to inconsistencies in preanalytical parameters (blood draws, plasma processing, *etc*.), which are known to influence EV analyses^[57–59]^. This highlights the importance of controlling and monitoring preanalytical conditions and incorporating quality controls during EV biomarker development. The development of preanalytical conditions or controls to overcome variability is a remaining challenge for the analytical validation of NDEV-based diagnostic assays, and we are actively working to overcome it.

Two biomarkers, proBDNF and GluR2, demonstrated significant differences between controls and early AD individuals across cohorts, which were maintained even after Bonferroni correction. Of note, these were also the only biomarkers that yielded significant correlations with MMSE and CDR-SOB alongside p181-Tau. ROC analysis pointed to p181-Tau as the best separator, as could be expected given its importance as a leading AD biomarker in all biofluids; however, its performance was closely followed by those of GluR2 and proBDNF, while GAP43, NRGN, Syntaxin-1, and PSD95 also showed significant AUC values. A model that combined multiple NDEV biomarkers accurately classified 94.7% of early AD patients but only 61.5% of controls. The lower classification accuracy for controls may be partly due to the lack of longitudinal follow-up, which may have led to mislabeling pre-symptomatic AD cases (as high as 10%−15% can be expected in this age group) as controls.

In summary, we introduce a novel methodology for NDEV isolation and a range of novel biomarkers for synaptic dysfunction in AD^[55,56]^. Despite limitations, e.g., the variance of absolute values for some analytes, we believe that the diagnostic promise of NDEVs and the method presented here were convincingly articulated. The development of effective AD treatments is costly and remains elusive, partly due to the lack of an efficient toolbox of minimally invasive blood biomarkers. Our results demonstrate the potential of novel NDE biomarkers reflecting synaptic dysfunction for diagnosis and, possibly, monitoring AD progression and treatment responses.

## Supplementary Material

supplementary

## Figures and Tables

**Table 1.** Demographic information for the two study cohorts

Cohort	NIA		BioIVT/PMED	
Sample group	Control	Early-stage AD	Control	Early-stage AD
Age	72.3 ± 6.97	73.53 ± 6.69	62.79 ± 6	66.22 ± 9.12
Gender	11F/9M	9F/10M	7F/12M	21F/19M
Mini mental state examination (MMSE) score	28.7 ± 2.05	26.42 ± 3.28	28.88 ± 1.05	23.87 ± 1.93
Clinical Dementia Rating Scale sum of boxes (CDR-SOB)	0	2.684 ± 1.5	0	3.98 ± 2.15

**Table 2.** TaqMan gene expression assays were used in the study

Gene ID	TaqMan assay ID (Thermo Fisher Scientific)
*HCRT*, hypocretin neuropeptide precursor/orexin	Hs01891339_s1
*NEFL*, Neurofilament Light Chain	Hs00196245_m1
*NRGN*, Neurogranin	Hs00382922_m1
*ENO2*, Enolase 2	Hs00157360_m1
*GPR26*, G protein-coupled receptor 26	Hs00538034_m1
*GPR101*, G protein-coupled receptor 101	Hs00369662_s1
*PSD95*, postsynaptic density protein 95	Hs01555373_m1

**Table 3.** Primary antibodies used in the study

Antigen	Vendor	CAT. No	Host	Type	Specificity
NRGN Neurogranin	Bio Legend	845702	Mouse	Monoclonal	Hu, Mo, Rat
NLGN Neuroligin 3	NeuroDex	NDXNL3	Rabbit	Monoclonal	Hu
Syntaxin-1 STXN1	BioLegend	827001	Mouse	Monoclonal	Hu, Mo, Rat
CD63-biotin CD63-bio	BioLegend	143918	Mouse	Monoclonal	Hu
CD81-biotin CD81-bio	BioLegend	349502	Mouse	Monoclonal	Hu
Rab3A Ras-like small GTPase 3A	Abcam	ab234089	Mouse	Monoclonal	Hu
CD171 L1 cell adhesion molecule (L1CAM)	Thermo Fisher Scientific	14-1719-82	Mouse	Monoclonal	Hu, Mo
PSD95 Postsynaptic density protein 95	BioLegend	810301	Mouse	Monoclonal	Hu, Mo, Rat
GluR1 AMPA receptor subunit 1	Abcam	ab183797	Rabbit	Monoclonal	Hu, Mo, Rat
CD9	Cell Signaling	13174S	Rabbit	Monoclonal	Hu
FLOT-1 Flotillin-1	Novus Biologicals	NBP1-79022	Rabbit	Polyclonal	Human
TSG101	Abcam	ab83	Mouse	Monoclonal	Human
GAP43 Growth-associated protein 43	NeuroDex	NDXGP43	Rabbit	Monoclonal	Hu, Mo, Rat
GluR2A Glutamate Receptor 2A	Thermo Fisher Scientific	MA5-17084	Mouse	Monoclonal	Hu, Mo, Rat
NeuN Neuron-specific nuclear protein 1	Abcam	ab177487	Rabbit	Monoclonal	Hu, Mo

**Table 4.** Measurement of neuronal markers in the NDEV isolation by ExoSORT and in control IgG pulldown

Analyte	NDEVs	IgG immunocapture	Fold increase
Protein (concentration)			
RGMa (Repulsive guidance molecule A)	2.3 ± 0.22 mg/ml	Below LLQ	N/A
Tau	458 ± 102 pg/ml	118 ± 20 pg/ml	4.05
Phospho-Tau (T181)	10 ± 1.4 pg/ml	3.9 ± 0.62 pg/ml	3.0
ProBDNF (Pro-Brain-Derived Neurotrophic Factor)	517 ± 17 pg/ml	150 ± 0.13 pg/ml	3.6
SYP (Synaptophysin)	1.8 ± 0.18 mg/ml	0.22 ± 0.02 mg/ml	10
NFL (Neurofilament light)	134 ± 17 pg/ml	16 ± 7 pg.ml	9.3
**Lipids (Concentration)**			
24-hydrohycholesterol	27 ± 2.7 ng/ml	Below LLQ	N/A
**RNA content (Ct value)**			
HCRT (hypothalamic neuropeptide precursor, Orexin)	26.4	31.7	39.4
NFL (Neurofilament light)	29.7	Below LLQ	N/A
NRGN (Neurogranin)	20.8	25.3	45.2
Eno2 (Enolase 2)	28	31.2	9.2
GPR26 (G protein-coupled receptor 26)	30.4	34.7	25.9
GPR101 (G protein-coupled receptor 101)	21.3	32.7	200
PSD95 (Presynaptic density protein)	35.1	Below LLQ	N/A

**Table 5.** Correlation analysis between NDEV biomarkers and clinical scores

		Ptau 181	NRGN	GAP43	PSD95	Syntaxin 1	SNAP25	GLUR2	proBDNF	MMSE	CDR.SOB
Ptau 181	Correlation	1	−0.71	−0.29	−0.288	−0.1	−0.163	−0.248	−0.113	−0.34	−0.396
	Significance (2-tailed)		0.496	0.004	0.005	0.333	0.122	0.015	0.274	0.001	0.008
	df	0	93	93	92	94	89	94	94	84	42
NRGN	Correlation	−0.71	1	0.135	0.297	0.137	0.652	0.066	−0.062	−0.009	0.117
	Significance (2-tailed)	0.496		0.194	0.004	0.184	0.001	0.524	0.549	0.934	0.455
	df	93	0	92	91	93	88	93	93	83	41
GAP43	Correlation	−0.29	0.135	1	0.645	0.585	0.204	0.359	0.045	0.097	0.197
	Significance (2-tailed)	0.004	0.194		0.001	0.001	0.045	0.001	0.663	0.375	0.204
	df	93	92	0	91	93	88	93	93	83	41
PSD95	Correlation	−0.288	0.297	0.645	1	0.775	0.264	0.578	0.021	0.024	0.26
	Significance (2-tailed)	0.005	0.004	0.001		0.001	0.012	0.001	0.841	0.825	0.096
	df	92	91	91	0	92	87	92	92	82	40
Syntaxin 1	Correlation	−0.1	0.137	0.585	0.775	1	0.159	0.598	0.219	0.073	0.122
	Significance (2-tailed)	0.333	0.184	0.001	0.001		0.132	0.001	0.032	0.504	0.43
	df	94	93	93	92	0	89	94	94	84	42
SNAP25	Correlation	−0.163	0.652	0.204	0.264	0.159	1	0.151	−0.025	−0.018	0.086
	Significance (2-tailed)	0.122	0.001	0.045	0.012	0.132		0.132	0.811	0.873	0.588
	df	89	88	88	87	89	0	89	89	79	40
GLUR2	Correlation	−0.248	0.066	0.359	0.578	0.598	0.151	1	0.105	0.225	0.543
	Significance (2-tailed)	0.015	0.524	0.001	0.001	0.001	0.132		0.307	0.037	0.001
	df	94	93	93	92	94	89	0	94	84	42
proBDNF	Correlation	−0.113	−0.062	0.045	0.021	0.219	−0.025	0.105	1	0.496	−0.308
	Significance (2-tailed)	0.274	0.549	0.663	0.841	0.032	0.811	0.307		0.001	0.042
	df	94	93	93	92	94	89	94	0	84	42
MMSE	Correlation	−0.34	−0.009	0.097	0.024	0.073	−0.018	0.225	0.496	1	−0.552
	Significance (2-tailed)	0.001	0.934	0.375	0.825	0.504	0.873	0.037	0.001		0.001
	df	84	83	83	82	84	79	84	84	0	42
CDR.SOB	Correlation	−0.396	0.117	0.197	0.26	0.122	0.086	0.543	−0.308	−0.552	1
	Significance (2-tailed)	0.008	0.455	0.204	0.096	0.43	0.588	0.001	0.042	0.001	
	df	42	41	41	40	42	40	42	42	42	0

## Data Availability

De-identified data will be made available to qualified investigators upon request.
