# Supplementary material for: Synaptic proteins in neuron-derived extracellular vesicles as biomarkers for Alzheimer’s disease: novel methodology and clinical proof of concept
Source: Extracell Vesicles Circ Nucl Acids. 2023 Mar 31;4(1):133–50. doi: 10.20517/evcna.2023.13 (PMC10568955; doi:10.20517/evcna.2023.13)
Supplement: Supplementary file 1 [file evcna-4-1-133-SupplementaryMaterials.pdf]

Supplementary Material

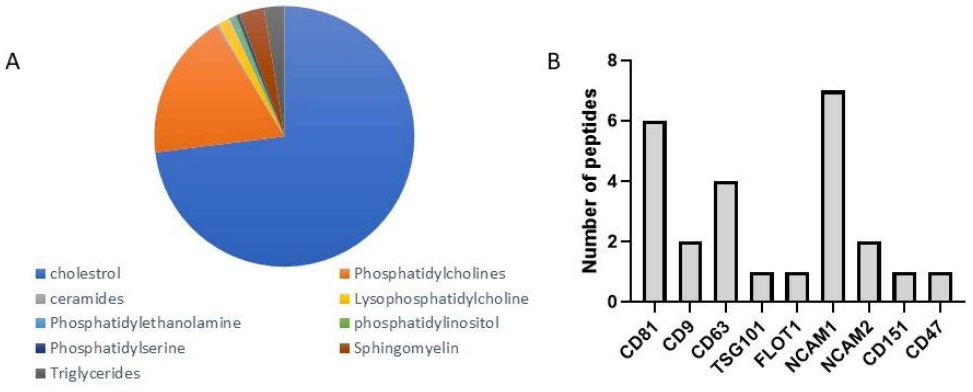

Supplementary Figure 1. Unbiased proteomic and lipidomic analysis confirms EV nature of material generated by ExoSORT.

Pooled plasma of 10 healthy donors was subjected to ExoSORT and isolated NDEs subjected to mass-spectrometry analysis for the lipid and protein composition. (**A**) A pie chart shows the major lipids constituents identified in the NDEs generated by ExoSORT. Not high percentage of cholesterol, sphingomyelin and ceramide are consistent with published EV lipidomic analyses. (**B**) Proteins reported previously as major EVs constituents were found by proteomic nalysis. A number of peptides detected is shown.

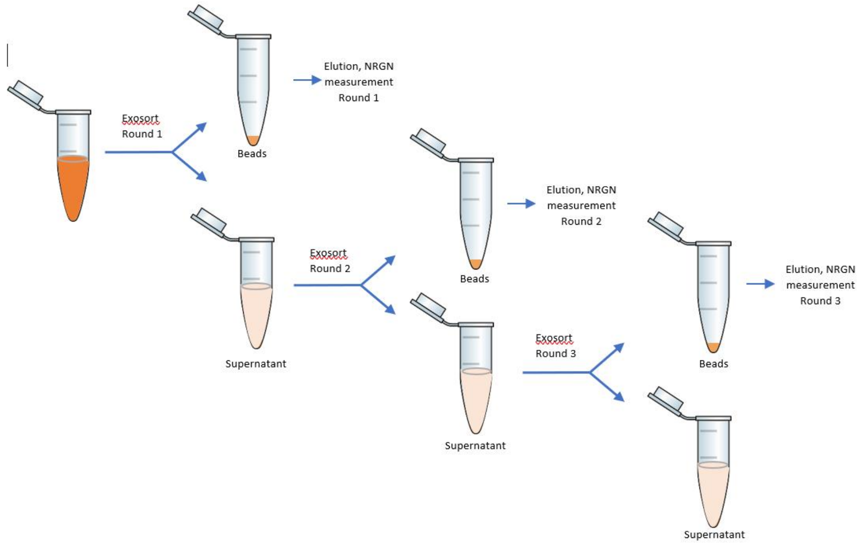

Supplementary Figure 2. Schematic representation of sequential isolation to estimate the efficiency of NDE recovery.

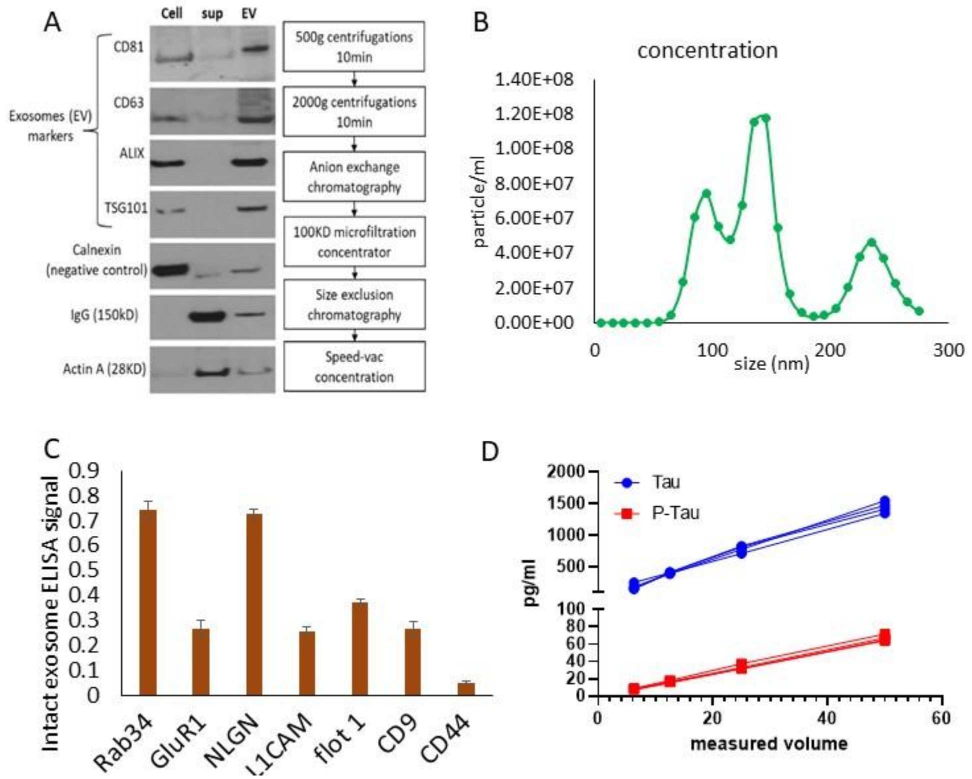

Supplementary Figure 3. EV isolation from the culture of IPS-derived cortical neurons.

Two hundred ml of conditioned media was purchased from BrainXell. The media were collected from the culture of IPS-derived cortical neurons was collected between weeks 4-7 of culture (differentiated state). EVs were isolated by a combination of ion exchange and size exclusion chromatography followed by a concentration step (see flow chart in panel **A** and Methods section). Before Isolation, IgG and actin A were spiked into the media to determine the extent of purification. (**A**) Western blot of EV lysates probed for EV markers CD81, CD63, Alix and TSG101, common cytoplasmic contaminant calnexin, and the spiked proteins (IgG, ActinA). Note high levels of EV markers and low contamination levels indicative of high purity. (**B**) The concentration and size distribution of the isolated EVs were determined by nanoparticle tracking analysis. (**C**) Neuronal proteins Rab3a, GLUR1, NLGN3 and L1CAM, as well as the EV markers Flot 1and CD9, as well as stem cells marker CD44 were measured by intact EV ELISA assay. (**D**) Tau and P181-Tau were measured in EV lysates using Milliplex assay.

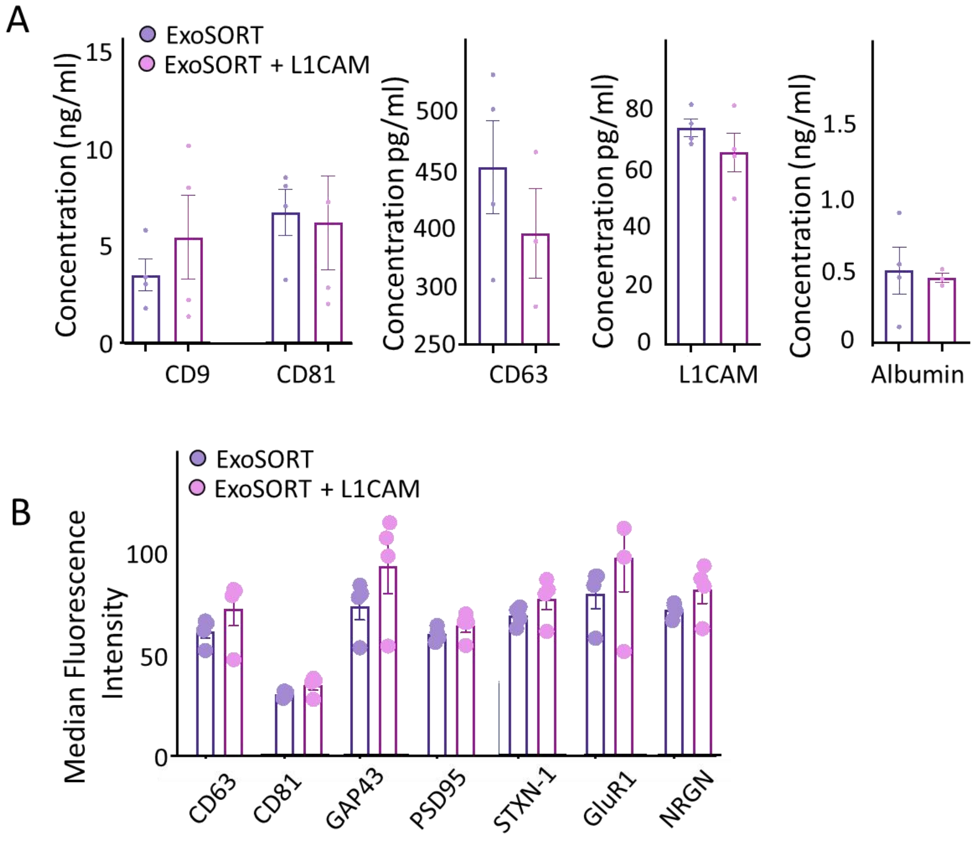

Supplementary Figure 4: Reproducibility of ExoSORT across independent labs/institutions

ExoSORT was reproduced in a second lab at the NIA (PI: D. Kapogianis). NeuroDex provided the ExoSORT kit to the NIA, where NDEs were isolated from plasma samples of four healthy controls by a proficient operator and compared to a modified protocol combining ExoSORT and L1CAM-based immunoprecipitation (IP). **A**. ExoSORT isolated NDEs express EV markers CD9, CD81 and CD63. D. ExoSORT isolated NDEs that also contain the neuronal protein CD171 (i.e., L1CAM), while adding anti-L1CAM antibody does not recover more L1CAM; ExoSORT with and without anti-L1CAM recover low levels of the serum protein albumin. **B**. Intact EV analysis also shows that ExoSORT with or without anti-L1CAM isolated NDEs that contain EV markers and synaptic proteins.

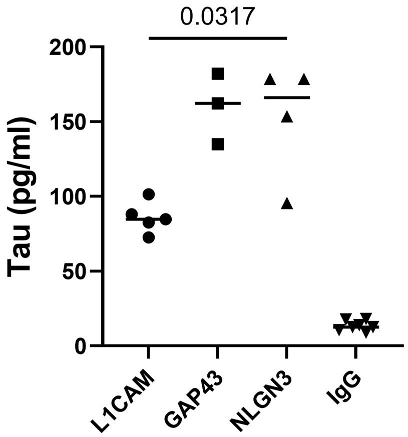

Supplementary Figure 5: Relative performance of three neuronal markers for NDE capture.

Multiple aliquots of the same plasma sample (pooled plasma from 10 disease-free donors) were subjected to ExoSORT procedure using antibodies against L1CAM, GAP43 and NLGN3 for EV capture. After elution and lysis, the levels of neuron-specific protein, Tau were evaluated using Milliplex kit from EMD Millipore. Non-specific isotype-matched antibodies were used as procedural control. Nota a statistically significant 2-fold increase in Tau levels when using GAP43 and NLGN3 antibodies.

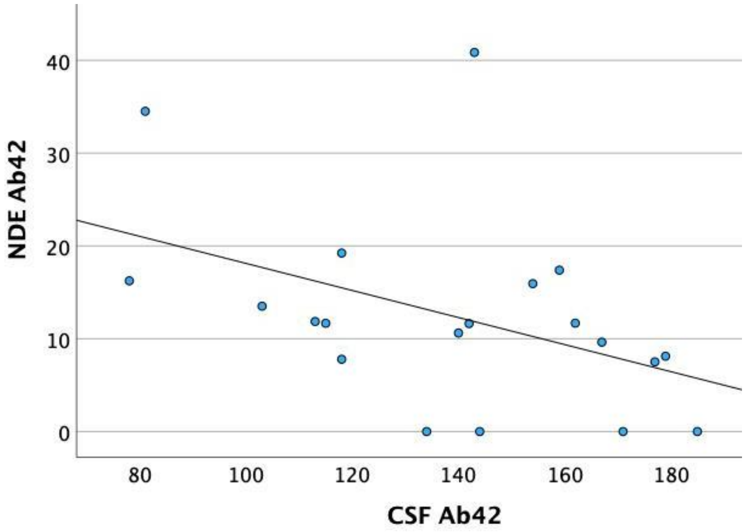

Supplementary Figure 6: Correlation analysis of p181Tau/Ab42 ratio in NDE and CSF.

For the NIH cohort, CSF was available for 16 out of 20 participants. For these individuals, Ab42 had a significant negative correlation with a Pearson correlation of -613 and a P value lower than 0.001 .

Supplementary Table 1A

	Concentration										Mol%									
											IgG	IgG	IgG	IgG	NDE	NDE	NDE	NDE	total EV	total EV
	AL 1-1	AL 1-2	AL 2-1	AL 2-2	AL 3-1	AL 3-2	AL 4-1	AL 4-2	AL 5-1	AL 5-2	AL 1-1	AL 1-2	AL 2-1	AL 2-2	AL 3-1	AL 3-2	AL 4-1	AL 4-2	AL 5-1	AL 5-2
**FC**	14.13028281	26.400719519	10.013449303	22.623813244	10.590135355	8.8216124	10.591794088	14.627197588	835.2407428	1295.800905	8.66	9.77	7.00	9.01	10.68	8.09	13.46	10.50	30.39	31.72
**CE**	108.4702169	169.0634109	97.080379911	154.1298079	58.796587232	69.837701299	43.177231878	80.067797899	1385.074894	2023.702049	66.48	62.59	67.83	61.41	59.31	64.02	54.87	57.47	50.40	49.54
**AC**	0.036202707	0.042873532	0.035996631	0.037378758	0.191055803	0.184198894	0.20038017	0.254125499	1.134222181	2.600139448	0.02	0.02	0.03	0.01	0.19	0.17	0.25	0.18	0.04	0.06
**MG**	0.176259252	0.184789764	0.174604384	0.26628815	0.868980531	0.845819669	0.802294806	1.445627569	0.762939642	1.711583912	0.11	0.07	0.12	0.11	0.88	0.78	1.02	1.04	0.03	0.04
**DG**	0.066389693	0.141955819	0.063502043	0.119972208	0.034686878	0.024793015	0.033827249	0.045607694	2.116939185	3.886169074	0.04	0.05	0.04	0.05	0.03	0.02	0.04	0.03	0.08	0.10
**TG**	8.321494695	17.60203787	9.12827504	16.9729918	3.190196065	2.714496461	2.193187644	3.939629424	56.39093887	99.21199198	5.10	6.52	6.38	6.76	3.22	2.49	2.79	2.83	2.05	2.43
**Cer**	0.110226377	0.154381271	0.104541823	0.143812893	0.098222356	0.10501259	0.100661145	0.144971106	2.907646995	6.706731208	0.07	0.06	0.07	0.06	0.10	0.10	0.13	0.10	0.11	0.16
**dh**	0.002	0.004	0.002	0.004	0.000	0.001	0.001	0.001	0.102	0.229	0.	0.	0.	0.	0.	0.	0.	0.	0.0	0.0

**Cer**	563257	161381	642942	241762	976381	678387	833047	773089	030629	269966		00	00	00	00	00	00	00	00	0	1	
**SM**	8.03636538	16.5381191	6.750338436	13.653377	3.454733662	3.241861229	2.554107729	4.178410786	140.6696121	221.0725678		4.93	6.12	4.72	5.44	3.49	2.97	3.25	3.00	5.12	5.41	
**dhSM**	0.081240146	0.169922663	0.07930984	0.157374644	0.062678965	0.048362961	0.041741805	0.094146067	5.838195087	12.01574261		0.05	0.06	0.06	0.06	0.06	0.04	0.05	0.07	0.21	0.29	
**MhCer**	0.107654485	0.143777757	0.11168668	0.129392908	0.107542704	0.128112713	0.103264378	0.118369047	2.068537254	3.824811825		0.07	0.05	0.08	0.05	0.11	0.12	0.13	0.08	0.08	0.09	
**Sulf**	0.014859967	0.021967982	0.016481351	0.015964704	0.019712371	0.022971639	0.014931309	0.024046967	0.233974634	0.51706222		0.01	0.01	0.01	0.01	0.02	0.02	0.02	0.02	0.01	0.01	
**LacCer**	0.069581384	0.117375497	0.041173225	0.111253117	0.033532578	0.04068958	0.013521125	0.023707302	5.306403058	10.43640757		0.04	0.04	0.03	0.04	0.03	0.04	0.02	0.02	0.19	0.26	
**GM3**	0.010148026	0.040717027	0.005230035	0.030855911	0.007337946	0.011134128	0.005277968	0.015779065	2.096273708	4.549889531		0.01	0.02	0.00	0.01	0.01	0.01	0.01	0.01	0.08	0.11	
**GB3**	0	0	0	0	0	0	0	0	0.529945632	0.831756791		0.00	0.00	0.00	0.00	0.00	0.00	0.00	0.00	0.02	0.02	
**PA**	0.033631786	0.032112033	0.029997979	0.03578097	0.029096598	0.03391849	0.030772028	0.034999397	0.130734761	0.094969443		0.02	0.01	0.02	0.01	0.03	0.03	0.04	0.03	0.00	0.00	
**PC**	21.1778982	35.3511559	17.5390003	38.2067617	16.5352279	17.8308680	13.9063534	26.07	191.422603	217.991364		12.9	13.0	12.2	15.2	16.6	16.3	17.6	18.7	6.9	5.3	

	5	7	6	8	3	7	5	0551	9	1		8	9	5	2	8	5	7	1	7	4	
**PCe**	1.826391998	2.947619945	1.403427147	3.322591532	1.606565113	1.808254152	1.365997373	2.612850838	34.80562078	40.20258507		1.12	1.09	0.98	1.32	1.62	1.66	1.74	1.88	1.27	0.98	
**PE**	0	0	0	0	0.0580311215	0.1101569411	0.0262067155	0.1590529977	14.01946144	21.64140775		0.00	0.00	0.00	0.00	0.06	0.10	0.03	0.11	0.51	0.53	
**PEp**	0.085826066	0.220991624	0.125857593	0.352711969	0.334201812	0.432773053	0.42819537	1.007805511	21.15850192	31.03950156		0.05	0.08	0.09	0.14	0.34	0.40	0.54	0.72	0.77	0.76	
**PS**	0.010054472	0.011270563	0.00835611	0.007465557	0.62511088	0.519325739	0.645736366	0.904259306	0.285746748	0.585817687		0.01	0.00	0.01	0.00	0.63	0.48	0.82	0.65	0.01	0.01	
**PI**	0.261719659	0.718584111	0.292111424	0.428301554	0.532688724	0.428533072	0.574485443	0.599446785	24.7251607	40.7965045		0.16	0.27	0.20	0.17	0.54	0.39	0.73	0.43	0.90	1.00	
**PG**	0.001710153	0.002981287	0.000937857	0.002106704	0.004810559	0.005014333	0.009400294	0.007786883	0.026286111	0.053692986		0.00	0.00	0.00	0.00	0.00	0.00	0.01	0.01	0.00	0.00	
**BMP**	0	0	0	0	0.00241284	0.003008639	0.00250589	0.00396292	0.023803651	0.060542629		0.00	0.00	0.00	0.00	0.00	0.00	0.00	0.00	0.00	0.00	
**AcylPG**	0	0	0	0	0	0	0	0	0	0		0.00	0.00	0.00	0.00	0.00	0.00	0.00	0.00	0.00	0.00	
**LPC**	0.09337348	0.17582532	0.06894218	0.15371956	1.5563646	1.62450635	1.44260074	2.36963958	17.7999349	39.2904676		0.06	0.07	0.05	0.06	1.57	1.49	1.83	1.70	0.65	0.96	

	9	4	7	7		3	1	5	7	3											
**LPCe**	0	0	0	0	0	0	0	0	0.403045662	0.982844895		0.00	0.00	0.00	0.00	0.00	0.00	0.00	0.00	0.01	0.02
**LPE**	0.02414658	0.027832432	0.036816537	0.058794451	0.27175088	0.169377744	0.323721543	0.399170805	2.831466548	4.939510938		0.01	0.01	0.03	0.02	0.27	0.16	0.41	0.29	0.10	0.12
**LPEp**	0	0	0	0	0	0	0	0	0	0		0.00	0.00	0.00	0.00	0.00	0.00	0.00	0.00	0.00	0.00
**LPI**	0.015212253	0.01482081	0.01309519	0.013338212	0.009936459	0.002740935	0.007191663	0.010802585	0.181703705	0.127165494		0.01	0.01	0.01	0.01	0.01	0.00	0.01	0.01	0.01	0.00
**LPS**	0	0	0	0	0.080087283	0.069655227	0.065874547	0.120618186	0.025204453	0.026203454		0.00	0.00	0.00	0.00	0.08	0.06	0.08	0.09	0.00	0.00
**NAPE**	0	0	0	0	0	0	0	0	0	0		0.00	0.00	0.00	0.00	0.00	0.00	0.00	0.00	0.00	0.00
**NAPS**	0	0	0	0	0	0	0	0	0.009192413	0.044074013		0.00	0.00	0.00	0.00	0.00	0.00	0.00	0.00	0.00	0.00
**NSer**	0	0	0	0	0.027642085	0.023501182	0.029237287	0.043088815	0.00366842	0.005413753		0.00	0.00	0.00	0.00	0.03	0.02	0.04	0.03	0.00	0.00

Supplementary Table 1B

Description	AvgOfPeptides
apolipoprotein B-100 [OS=Homo sapiens]	89.14285714
fibronectin [OS=Homo sapiens]	52.85714286
alpha-2-macroglobulin [OS=Homo sapiens]	42.42857143
Keratin, type II cytoskeletal 1 [OS=Homo sapiens]	39
Keratin, type II cytoskeletal 2 epidermal [OS=Homo sapiens]	37.28571429
Serum albumin [OS=Homo sapiens]	35.28571429
Complement C4-B [OS=Homo sapiens]	31.71428571
Complement C4-A [OS=Homo sapiens]	31.14285714
Desmoplakin [OS=Homo sapiens]	30.57142857
complement factor H [OS=Homo sapiens]	29
Serotransferrin [OS=Homo sapiens]	28.85714286
keratin, type II cytoskeletal 5 [OS=Homo sapiens]	28.57142857
Keratin, type I cytoskeletal 9 [OS=Homo sapiens]	27.42857143
Keratin, type I cytoskeletal 10 [OS=Homo sapiens]	25.28571429
Plasma kallikrein [OS=Homo sapiens]	24.57142857
Fibrinogen alpha chain [OS=Homo sapiens]	24.14285714
Keratin, type I cytoskeletal 14 [OS=Homo sapiens]	23.28571429
Keratin, type II cytoskeletal 6A [OS=Homo sapiens]	23
keratin, type II cytoskeletal 6B [OS=Homo sapiens]	23
Fibrinogen beta chain [OS=Homo sapiens]	22.42857143
Keratin, type II cytoskeletal 6C [OS=Homo sapiens]	22

Plasminogen [OS=Homo sapiens]	21.28571429
Coagulation factor V [OS=Homo sapiens]	20
Fibrinogen gamma chain [OS=Homo sapiens]	18.71428571
Keratin, type II cuticular Hb6 [OS=Homo sapiens]	18
Keratin, type I cytoskeletal 16 [OS=Homo sapiens]	17.71428571
Haptoglobin [OS=Homo sapiens]	17.71428571
Apolipoprotein A-I [OS=Homo sapiens]	16.85714286
kininogen-1 [OS=Homo sapiens]	16.5
keratin, type II cuticular Hb3 [OS=Homo sapiens]	16
Ceruloplasmin [OS=Homo sapiens]	15.85714286
Complement C5 [OS=Homo sapiens]	15
Keratin, type I cuticular Ha1 [OS=Homo sapiens]	15
Isoform LMW of Kininogen-1 [OS=Homo sapiens]	15
immunoglobulin gamma-1 heavy chain [OS=Homo sapiens]	15
Keratin, type II cytoskeletal 75 [OS=Homo sapiens]	15
Apolipoprotein E [OS=Homo sapiens]	14.57142857
Von Willebrand factor [OS=Homo sapiens]	14.5
apolipoprotein(a) [OS=Homo sapiens]	14.42857143
Keratin, type I cytoskeletal 17 [OS=Homo sapiens]	13.85714286
Tenascin [OS=Homo sapiens]	13.33333333
immunoglobulin heavy constant mu [OS=Homo sapiens]	13.28571429
Keratin, type I cytoskeletal 13 [OS=Homo sapiens]	12.8

Immunoglobulin heavy constant gamma 3 [OS=Homo sapiens]	12.71428571
Inter-alpha-trypsin inhibitor heavy chain H2 [OS=Homo sapiens]	12.57142857
alpha-1-antitrypsin [OS=Homo sapiens]	12.28571429
Isoform 2 of Dynamin-1 [OS=Homo sapiens]	12
Keratin, type II cytoskeletal 3 [OS=Homo sapiens]	12
Coagulation factor XI [OS=Homo sapiens]	11.5
immunoglobulin heavy constant alpha 1 [OS=Homo sapiens]	11.42857143
Isoform 1B of Desmocollin-1 [OS=Homo sapiens]	11
Immunoglobulin heavy constant gamma 1 [OS=Homo sapiens]	11
Keratin, type II cytoskeletal 4 [OS=Homo sapiens]	11
Keratin, type II cuticular Hb5 [OS=Homo sapiens]	11
Hornerin [OS=Homo sapiens]	10.71428571
Protein kinase C-binding protein NELL2 [OS=Homo sapiens]	10.5
Haptoglobin-related protein [OS=Homo sapiens]	10.33333333
Immunoglobulin heavy constant gamma 2 [OS=Homo sapiens]	10.28571429
Actin, cytoplasmic 1 [OS=Homo sapiens]	10.14285714
Keratin, type II cytoskeletal 2 oral [OS=Homo sapiens]	10
Keratin, type I cuticular Ha3-II [OS=Homo sapiens]	10
keratin, type I cuticular Ha4 [OS=Homo sapiens]	10
vitamin D-binding protein [OS=Homo sapiens]	9.857142857
Coagulation factor XIII A chain [OS=Homo sapiens]	9.714285714

Complement factor B [OS=Homo sapiens]	9.571428571
Keratin, type II cytoskeletal 1b [OS=Homo sapiens]	9.571428571
Immunoglobulin heavy constant gamma 4 [OS=Homo sapiens]	9.285714286
Immunoglobulin alpha-2 heavy chain [OS=Homo sapiens]	9.25
Heat shock protein HSP 90-alpha [OS=Homo sapiens]	9.166666667
Agrin [OS=Homo sapiens]	9
Keratin, type II cytoskeletal 79 [OS=Homo sapiens]	9
Brevican core protein [OS=Homo sapiens]	8.8
C4b-binding protein alpha chain [OS=Homo sapiens]	8.714285714
Receptor-type tyrosine-protein phosphatase zeta [OS=Homo sapiens]	8.5
Transitional endoplasmic reticulum ATPase [OS=Homo sapiens]	8.4
Coagulation factor XIII B chain [OS=Homo sapiens]	8.285714286
Clusterin [OS=Homo sapiens]	8.285714286
Hemopexin [OS=Homo sapiens]	8.285714286
Histidine-rich glycoprotein [OS=Homo sapiens]	8.142857143
Apolipoprotein A-IV [OS=Homo sapiens]	8.142857143
Keratin, type I cytoskeletal 15 [OS=Homo sapiens]	8
Pregnancy zone protein [OS=Homo sapiens]	8
Versican core protein [OS=Homo sapiens]	8
Glycogen phosphorylase, brain form [OS=Homo sapiens]	8
Inter-alpha-trypsin inhibitor heavy chain H4 [OS=Homo sapiens]	7.857142857

Complement C1r subcomponent [OS=Homo sapiens]	7.833333333
fructose-bisphosphate aldolase A [OS=Homo sapiens]	7.8
Neurocan core protein [OS=Homo sapiens]	7.75
actin, gamma-enteric smooth muscle [OS=Homo sapiens]	7.666666667
Heat shock cognate 71 kDa protein [OS=Homo sapiens]	7.6
immunoglobulin kappa light chain [OS=Homo sapiens]	7.428571429
Desmoglein-1 [OS=Homo sapiens]	7.428571429
Heat shock protein HSP 90-beta [OS=Homo sapiens]	7.4
Filaggrin-2 [OS=Homo sapiens]	7.285714286
Keratin, type II cytoskeletal 78 [OS=Homo sapiens]	7.285714286
Dynamin-2 [OS=Homo sapiens]	7
Junction plakoglobin [OS=Homo sapiens]	6.857142857
Pyruvate kinase PKM [OS=Homo sapiens]	6.833333333
Prothrombin [OS=Homo sapiens]	6.8
Clathrin heavy chain 1 [OS=Homo sapiens]	6.666666667
14-3-3 protein epsilon [OS=Homo sapiens]	6.6
immunoglobulin kappa constant [OS=Homo sapiens]	6.571428571
Rab GDP dissociation inhibitor alpha [OS=Homo sapiens]	6.5
Sodium/potassium-transporting ATPase subunit alpha-3 [OS=Homo sapiens]	6.5
alpha-enolase [OS=Homo sapiens]	6.285714286
Malate dehydrogenase, mitochondrial [OS=Homo sapiens]	6.25
14-3-3 protein beta/alpha [OS=Homo sapiens]	6.2

14-3-3 protein zeta/delta [OS=Homo sapiens]	6.166666667
Complement factor I [OS=Homo sapiens]	6.142857143
Keratin, type I cytoskeletal 19 [OS=Homo sapiens]	6
Synaptotagmin-1 [OS=Homo sapiens]	6
Dihydropyrimidinase-related protein 2 [OS=Homo sapiens]	6
Syntaxin-binding protein 1 [OS=Homo sapiens]	6
Suprabasin [OS=Homo sapiens]	6
Synaptic vesicle membrane protein VAT-1 homolog-like [OS=Homo sapiens]	6
Isoform 2 of Extracellular matrix protein 1 [OS=Homo sapiens]	6
Keratin, type II cytoskeletal 80 [OS=Homo sapiens]	5.857142857
Complement factor H-related protein 1 [OS=Homo sapiens]	5.857142857
14-3-3 protein theta [OS=Homo sapiens]	5.8
Dynamin-1 [OS=Homo sapiens]	5.75
thrombospondin-1 [OS=Homo sapiens]	5.75
Vitamin K-dependent protein S [OS=Homo sapiens]	5.75
Antithrombin-III [OS=Homo sapiens]	5.714285714
Vimentin [OS=Homo sapiens]	5.666666667
Immunoglobulin heavy constant alpha 2 [OS=Homo sapiens]	5.666666667
Gamma-enolase [OS=Homo sapiens]	5.6
tubulin alpha-1A chain [OS=Homo sapiens]	5.5
Isoform 2 of Spectrin alpha chain, non-erythrocytic 1 [OS=Homo sapiens]	5.5

14-3-3 protein eta [OS=Homo sapiens]	5.4
Endoplasmin [OS=Homo sapiens]	5.4
Creatine kinase B-type [OS=Homo sapiens]	5.333333333
Neuronal cell adhesion molecule [OS=Homo sapiens]	5.333333333
Complement C1s subcomponent [OS=Homo sapiens]	5.285714286
CD5 antigen-like [OS=Homo sapiens]	5.285714286
Fructose-bisphosphate aldolase C [OS=Homo sapiens]	5.25
peroxiredoxin-1 [OS=Homo sapiens]	5.25
Immunoglobulin lambda-1 light chain [OS=Homo sapiens]	5.2
Heparin cofactor 2 [OS=Homo sapiens]	5.166666667
Sodium/potassium-transporting ATPase subunit alpha-2 [OS=Homo sapiens]	5
Serpin B3 [OS=Homo sapiens]	5
Serpin A12 [OS=Homo sapiens]	5
Stress-70 protein, mitochondrial [OS=Homo sapiens]	5
thyroxine-binding globulin [OS=Homo sapiens]	5
Tubulin beta-4B chain [OS=Homo sapiens]	5
Phosphoglucomutase-1 [OS=Homo sapiens]	5
phosphoglycerate kinase 1 [OS=Homo sapiens]	5
Isoform 6 of Dynamin-1-like protein [OS=Homo sapiens]	5
ATP synthase subunit beta, mitochondrial [OS=Homo sapiens]	5
Afamin [OS=Homo sapiens]	5
tubulin beta-3 chain [OS=Homo sapiens]	5

Selenium-binding protein 1 [OS=Homo sapiens]	5
Keratin, type II cytoskeletal 7 [OS=Homo sapiens]	5
1-phosphatidylinositol 4,5-bisphosphate phosphodiesterase beta-1 [OS=Homo sapiens]	5
14-3-3 protein gamma [OS=Homo sapiens]	5
Immunoglobulin lambda-like polypeptide 5 [OS=Homo sapiens]	5
60 kDa heat shock protein, mitochondrial [OS=Homo sapiens]	4.8
Galectin-3-binding protein [OS=Homo sapiens]	4.8
Complement component c6 [OS=Homo sapiens]	4.714285714
Inter-alpha-trypsin inhibitor heavy chain H1 [OS=Homo sapiens]	4.714285714
apolipoprotein D [OS=Homo sapiens]	4.714285714
Serine/threonine-protein phosphatase 2B catalytic subunit alpha isoform [OS=Homo sapiens]	4.666666667
Myristoylated alanine-rich C-kinase substrate [OS=Homo sapiens]	4.666666667
Complement factor H-related protein 5 [OS=Homo sapiens]	4.666666667
Contactin-1 [OS=Homo sapiens]	4.666666667
Isoform 2 of Neural cell adhesion molecule 1 [OS=Homo sapiens]	4.666666667
Isoform 2 of Suprabasin [OS=Homo sapiens]	4.666666667
Alpha-1-antichymotrypsin [OS=Homo sapiens]	4.571428571
Serum paraoxonase/arylesterase 1 [OS=Homo sapiens]	4.571428571
Filaggrin [OS=Homo sapiens]	4.5
Ras-related protein Rab-3A [OS=Homo sapiens]	4.5

Keratin, type II cytoskeletal 72 [OS=Homo sapiens]	4.5
Calsyntenin-1 [OS=Homo sapiens]	4.5
Dihydropyrimidinase-related protein 1 [OS=Homo sapiens]	4.4
Alpha-1B-glycoprotein [OS=Homo sapiens]	4.4
L-lactate dehydrogenase B chain [OS=Homo sapiens]	4.4
Hemoglobin subunit beta [OS=Homo sapiens]	4.333333333
tubulin beta chain [OS=Homo sapiens]	4.333333333
glyceraldehyde-3-phosphate dehydrogenase [OS=Homo sapiens]	4.333333333
Brain acid soluble protein 1 [OS=Homo sapiens]	4.333333333
Vitronectin [OS=Homo sapiens]	4.285714286
Protein AMBP [OS=Homo sapiens]	4.285714286
Serum amyloid P-component [OS=Homo sapiens]	4.285714286
Complement component C7 [OS=Homo sapiens]	4.285714286
Beta-enolase [OS=Homo sapiens]	4.25
Apolipoprotein L1 [OS=Homo sapiens]	4.166666667
Immunoglobulin J chain [OS=Homo sapiens]	4.142857143
complement component C9 [OS=Homo sapiens]	4.142857143
nucleoside diphosphate kinase b [OS=Homo sapiens]	4
Keratin, type I cytoskeletal 27 [OS=Homo sapiens]	4
Keratin, type I cuticular Ha6 [OS=Homo sapiens]	4
dihydropteridine reductase [OS=Homo sapiens]	4
immunoglobulin lambda constant 2 [OS=Homo sapiens]	4

immunoglobulin heavy variable 3-73 [OS=Homo sapiens]	4
Glutamate dehydrogenase 1, mitochondrial [OS=Homo sapiens]	4
Ras-related protein Rab-1A [OS=Homo sapiens]	4
ras-related protein Rab-3C [OS=Homo sapiens]	4
ras-related protein Rab-3D [OS=Homo sapiens]	4
Isocitrate dehydrogenase [NAD] subunit alpha, mitochondrial [OS=Homo sapiens]	4
ras-related protein Rab-7a [OS=Homo sapiens]	4
Endophilin-A1 [OS=Homo sapiens]	4
Heterogeneous nuclear ribonucleoprotein K [OS=Homo sapiens]	4
Vesicle-fusing ATPase [OS=Homo sapiens]	4
serine/threonine-protein phosphatase 2A 65 kDa regulatory subunit A alpha isoform [OS=Homo sapiens]	4
Aspartate aminotransferase, mitochondrial [OS=Homo sapiens]	4
serine/threonine-protein phosphatase 2A catalytic subunit beta isoform [OS=Homo sapiens]	4
Synapsin-2 [OS=Homo sapiens]	4
Syntaxin-1B [OS=Homo sapiens]	4
Spectrin beta chain, non-erythrocytic 1 [OS=Homo sapiens]	4
Cofilin-2 [OS=Homo sapiens]	4
V-type proton ATPase subunit E 1 [OS=Homo sapiens]	4
Complement C1q subcomponent subunit C [OS=Homo sapiens]	3.857142857
Complement C1q subcomponent subunit B [OS=Homo sapiens]	3.8

Alpha-1-acid glycoprotein 1 [OS=Homo sapiens]	3.714285714
14-3-3 protein sigma [OS=Homo sapiens]	3.666666667
Complement factor H-related protein 2 [OS=Homo sapiens]	3.666666667
Protein kinase C and casein kinase substrate in neurons protein 1 [OS=Homo sapiens]	3.666666667
Tubulin beta-2A chain [OS=Homo sapiens]	3.666666667
Protein-glutamine gamma-glutamyltransferase E [OS=Homo sapiens]	3.571428571
Hexokinase-1 [OS=Homo sapiens]	3.5
AP-2 complex subunit beta [OS=Homo sapiens]	3.5
Immunoglobulin heavy variable 3-7 [OS=Homo sapiens]	3.5
Attractin [OS=Homo sapiens]	3.5
Ubiquitin-like modifier-activating enzyme 1 [OS=Homo sapiens]	3.5
Glial fibrillary acidic protein [OS=Homo sapiens]	3.5
Clathrin coat assembly protein AP180 [OS=Homo sapiens]	3.5
Extracellular matrix protein 1 [OS=Homo sapiens]	3.5
immunoglobulin heavy variable 3-74 [OS=Homo sapiens]	3.4
Annexin A2 [OS=Homo sapiens]	3.4
Immunoglobulin heavy variable 3-15 [OS=Homo sapiens]	3.333333333
Cathepsin D [OS=Homo sapiens]	3.333333333
Isoform 2 of Triosephosphate isomerase [OS=Homo sapiens]	3.333333333
Synapsin-1 [OS=Homo sapiens]	3.333333333

V-type proton ATPase catalytic subunit A [OS=Homo sapiens]	3.333333333
V-type proton ATPase subunit B, brain isoform [OS=Homo sapiens]	3.333333333
lysozyme c [OS=Homo sapiens]	3.285714286
Alpha-2-HS-glycoprotein [OS=Homo sapiens]	3.25
Aconitate hydratase, mitochondrial [OS=Homo sapiens]	3.25
Immunoglobulin heavy variable 3-30 [OS=Homo sapiens]	3.2
Plasma protease C1 inhibitor [OS=Homo sapiens]	3.166666667
78 kDa glucose-regulated protein [OS=Homo sapiens]	3.166666667
immunoglobulin heavy variable 3-72 [OS=Homo sapiens]	3.166666667
Fatty acid-binding protein, epidermal [OS=Homo sapiens]	3.142857143
AP-2 complex subunit alpha-1 [OS=Homo sapiens]	3
Tropomyosin alpha-3 chain [OS=Homo sapiens]	3
Inter-alpha-trypsin inhibitor heavy chain H3 [OS=Homo sapiens]	3
Plakophilin-1 [OS=Homo sapiens]	3
Transgelin-3 [OS=Homo sapiens]	3
Arginase-1 [OS=Homo sapiens]	3
Immunoglobulin lambda variable 7-43 [OS=Homo sapiens]	3
Immunoglobulin lambda variable 3-9 [OS=Homo sapiens]	3
immunoglobulin lambda constant 7 [OS=Homo sapiens]	3
Poly(RC)-binding protein 1 [OS=Homo sapiens]	3

Beta-2-glycoprotein 1 [OS=Homo sapiens]	3
High mobility group protein B1 [OS=Homo sapiens]	3
Thioredoxin-like protein 1 [OS=Homo sapiens]	3
coronin-1C [OS=Homo sapiens]	3
prolow-density lipoprotein receptor-related protein 1 [OS=Homo sapiens]	3
eukaryotic initiation factor 4A-II [OS=Homo sapiens]	3
Cytoplasmic FMR1-interacting protein 2 [OS=Homo sapiens]	3
Proteasome subunit alpha type-1 [OS=Homo sapiens]	3
Immunoglobulin heavy variable 3-33 [OS=Homo sapiens]	3
proteasome subunit beta type-1 [OS=Homo sapiens]	3
Elongation factor 2 [OS=Homo sapiens]	3
Calbindin [OS=Homo sapiens]	3
immunoglobulin delta heavy chain [OS=Homo sapiens]	3
Hypoxanthine-guanine phosphoribosyltransferase [OS=Homo sapiens]	3
Histone H2B type 2-F [OS=Homo sapiens]	3
ATP-dependent 6-phosphofructokinase, muscle type [OS=Homo sapiens]	3
Septin-7 [OS=Homo sapiens]	3
Myelin proteolipid protein [OS=Homo sapiens]	3
Myc box-dependent-interacting protein 1 [OS=Homo sapiens]	3
Multiple epidermal growth factor-like domains protein 8 [OS=Homo sapiens]	3

X-ray repair cross-complementing protein 6 [OS=Homo sapiens]	3
Malate dehydrogenase, cytoplasmic [OS=Homo sapiens]	3
lipopolysaccharide-binding protein [OS=Homo sapiens]	3
Neurochondrin [OS=Homo sapiens]	3
LIM and SH3 domain protein 1 [OS=Homo sapiens]	3
Lactotransferrin [OS=Homo sapiens]	3
V-type proton ATPase subunit H [OS=Homo sapiens]	3
Coronin-1A [OS=Homo sapiens]	3
Keratin, type II cytoskeletal 74 [OS=Homo sapiens]	3
Isoform 3B of Desmocollin-3 [OS=Homo sapiens]	3
Keratin, type II cytoskeletal 71 [OS=Homo sapiens]	3
NIF3-like protein 1 [OS=Homo sapiens]	3
decorin [OS=Homo sapiens]	3
nuclear migration protein nudC [OS=Homo sapiens]	3
4-aminobutyrate aminotransferase, mitochondrial [OS=Homo sapiens]	3
ADP-ribosylation factor 5 [OS=Homo sapiens]	3
cullin-associated nedd8-dissociated protein 1 [OS=Homo sapiens]	3
Alpha-1-acid glycoprotein 2 [OS=Homo sapiens]	3
Alpha-synuclein [OS=Homo sapiens]	3
aminopeptidase B [OS=Homo sapiens]	3
immunoglobulin kappa variable 3D-20 [OS=Homo sapiens]	3

26S proteasome regulatory subunit 7 [OS=Homo sapiens]	3
Glia-derived nexin [OS=Homo sapiens]	3
serine/threonine-protein phosphatase PP1-alpha catalytic subunit [OS=Homo sapiens]	3
Glycogen phosphorylase, liver form [OS=Homo sapiens]	3
puromycin-sensitive aminopeptidase [OS=Homo sapiens]	3
Guanine nucleotide-binding protein G(I)/G(S)/G(T) subunit beta-2 [OS=Homo sapiens]	3
Complement component C8 alpha chain [OS=Homo sapiens]	3
Hemoglobin subunit delta [OS=Homo sapiens]	3
Carboxypeptidase B2 [OS=Homo sapiens]	3
Pyruvate dehydrogenase E1 component subunit alpha, somatic form, mitochondrial [OS=Homo sapiens]	3
splicing factor, proline- and glutamine-rich [OS=Homo sapiens]	3
fumarate hydratase, mitochondrial [OS=Homo sapiens]	3
Sex hormone-binding globulin [OS=Homo sapiens]	3
complement C2 [OS=Homo sapiens]	3
Heterogeneous nuclear ribonucleoproteins C1/C2 [OS=Homo sapiens]	3
Coagulation factor XII [OS=Homo sapiens]	3
Gelsolin [OS=Homo sapiens]	2.857142857
Protein-glutamine gamma-glutamyltransferase K [OS=Homo sapiens]	2.857142857
Keratinocyte proline-rich protein [OS=Homo sapiens]	2.833333333
Caspase-14 [OS=Homo sapiens]	2.833333333

Alpha-2-antiplasmin [OS=Homo sapiens]	2.833333333
Desmocollin-1 [OS=Homo sapiens]	2.8
Selenoprotein P [OS=Homo sapiens]	2.75
bleomycin hydrolase [OS=Homo sapiens]	2.714285714
immunoglobulin heavy variable 5-51 [OS=Homo sapiens]	2.714285714
Hemoglobin subunit alpha [OS=Homo sapiens]	2.714285714
Immunoglobulin kappa variable 3-20 [OS=Homo sapiens]	2.714285714
Glypican-1 [OS=Homo sapiens]	2.666666667
Dihydropyrimidinase-related protein 3 [OS=Homo sapiens]	2.666666667
cholesteryl ester transfer protein [OS=Homo sapiens]	2.666666667
Cystatin-A [OS=Homo sapiens]	2.666666667
Calmodulin-like protein 5 [OS=Homo sapiens]	2.666666667
Aspartate aminotransferase, cytoplasmic [OS=Homo sapiens]	2.666666667
Mannan-binding lectin serine protease 1 [OS=Homo sapiens]	2.6
Keratin, type I cytoskeletal 23 [OS=Homo sapiens]	2.6
immunoglobulin lambda variable 3-10 [OS=Homo sapiens]	2.6
immunoglobulin heavy variable 4-34 [OS=Homo sapiens]	2.6
Immunoglobulin kappa variable 2-29 [OS=Homo sapiens]	2.571428571
Fibulin-1 [OS=Homo sapiens]	2.571428571
Transthyretin [OS=Homo sapiens]	2.571428571

immunoglobulin heavy variable 3-49 [OS=Homo sapiens]	2.571428571
T-complex protein 1 subunit gamma [OS=Homo sapiens]	2.5
Protein tweety homolog 1 [OS=Homo sapiens]	2.5
Prelamin-A/C [OS=Homo sapiens]	2.5
BTB/POZ domain-containing protein 17 [OS=Homo sapiens]	2.5
Small proline-rich protein 3 [OS=Homo sapiens]	2.5
Laminin subunit gamma-1 [OS=Homo sapiens]	2.5
Retinol-binding protein 4 [OS=Homo sapiens]	2.5
Zinc-alpha-2-glycoprotein [OS=Homo sapiens]	2.5
angiogenin [OS=Homo sapiens]	2.5
Calretinin [OS=Homo sapiens]	2.5
Cofilin-1 [OS=Homo sapiens]	2.5
Acylamino-acid-releasing enzyme [OS=Homo sapiens]	2.5
T-complex protein 1 subunit theta [OS=Homo sapiens]	2.5
Keratin, type I cytoskeletal 20 [OS=Homo sapiens]	2.5
Proteasome subunit alpha type-4 [OS=Homo sapiens]	2.5
mitogen-activated protein kinase 1 [OS=Homo sapiens]	2.5
ADP-ribosylation factor 3 [OS=Homo sapiens]	2.5
Proteasome subunit alpha type-7 [OS=Homo sapiens]	2.4
Immunoglobulin kappa variable 2-30 [OS=Homo sapiens]	2.4
Immunoglobulin heavy variable 3-43 [OS=Homo sapiens]	2.333333333

Isocitrate dehydrogenase [NADP] cytoplasmic [OS=Homo sapiens]	2.333333333
Neuronal membrane glycoprotein M6-a [OS=Homo sapiens]	2.333333333
WD repeat-containing protein 1 [OS=Homo sapiens]	2.333333333
Hyaluronan-binding protein 2 [OS=Homo sapiens]	2.333333333
Pro-epidermal growth factor [OS=Homo sapiens]	2.333333333
stress-induced-phosphoprotein 1 [OS=Homo sapiens]	2.333333333
Beta-synuclein [OS=Homo sapiens]	2.333333333
Alpha-actinin-4 [OS=Homo sapiens]	2.333333333
cholinesterase [OS=Homo sapiens]	2.333333333
Apolipoprotein C-I [OS=Homo sapiens]	2.285714286
Immunoglobulin heavy variable 3-43D [OS=Homo sapiens]	2.285714286
Phosphoglycerate mutase 1 [OS=Homo sapiens]	2.25
Isoform 1 of Protein POF1B [OS=Homo sapiens]	2.25
Ubiquitin carboxyl-terminal hydrolase isozyme L1 [OS=Homo sapiens]	2.25
immunoglobulin heavy variable 5-10-1 [OS=Homo sapiens]	2.25
Calmodulin [OS=Homo sapiens]	2.25
immunoglobulin heavy variable 4-4 [OS=Homo sapiens]	2.2
Amyloid-beta A4 protein [OS=Homo sapiens]	2.2
Serpin B12 [OS=Homo sapiens]	2.2
catalase [OS=Homo sapiens]	2.166666667
immunoglobulin heavy variable 4-28 [OS=Homo sapiens]	2.142857143

Apolipoprotein C-II [OS=Homo sapiens]	2.142857143
Elongation factor 1-alpha 1 [OS=Homo sapiens]	2
26S proteasome non-ATPase regulatory subunit 2 [OS=Homo sapiens]	2
Immunoglobulin heavy variable 3-13 [OS=Homo sapiens]	2
clathrin light chain B [OS=Homo sapiens]	2
Hepatocyte growth factor-regulated tyrosine kinase substrate [OS=Homo sapiens]	2
40S ribosomal protein S14 [OS=Homo sapiens]	2
Gamma-synuclein [OS=Homo sapiens]	2
caM kinase-like vesicle-associated protein [OS=Homo sapiens]	2
Elongation factor 1-alpha 2 [OS=Homo sapiens]	2
2',3'-cyclic-nucleotide 3'-phosphodiesterase [OS=Homo sapiens]	2
40S ribosomal protein S19 [OS=Homo sapiens]	2
40S ribosomal protein S3a [OS=Homo sapiens]	2
Immunoglobulin heavy variable 4-30-2 [OS=Homo sapiens]	2
Delta-1-pyrroline-5-carboxylate dehydrogenase, mitochondrial [OS=Homo sapiens]	2
Actin-related protein 2 [OS=Homo sapiens]	2
elongation factor 1-gamma [OS=Homo sapiens]	2
cytosolic acyl coenzyme A thioester hydrolase [OS=Homo sapiens]	2
Myotrophin [OS=Homo sapiens]	2
Collagen alpha-2(I) chain [OS=Homo sapiens]	2

cold shock domain-containing protein E1 [OS=Homo sapiens]	2
Histidine ammonia-lyase [OS=Homo sapiens]	2
Moesin [OS=Homo sapiens]	2
histone H4 [OS=Homo sapiens]	2
microtubule-associated protein 2 [OS=Homo sapiens]	2
Elongin-B [OS=Homo sapiens]	2
Immunoglobulin heavy variable 1-3 [OS=Homo sapiens]	2
Macrophage colony-stimulating factor 1 [OS=Homo sapiens]	2
Immunoglobulin heavy variable 1-18 [OS=Homo sapiens]	2
Lumican [OS=Homo sapiens]	2
D-aminoacyl-tRNA deacylase 1 [OS=Homo sapiens]	2
L-lactate dehydrogenase A chain [OS=Homo sapiens]	2
Calcyclin-binding protein [OS=Homo sapiens]	2
Heterogeneous nuclear ribonucleoprotein A3 [OS=Homo sapiens]	2
C4b-binding protein beta chain [OS=Homo sapiens]	2
Coagulation factor X [OS=Homo sapiens]	2
BPI fold-containing family B member 1 [OS=Homo sapiens]	2
biglycan [OS=Homo sapiens]	2
Heterogeneous nuclear ribonucleoprotein L [OS=Homo sapiens]	2
Insulin-like growth factor-binding protein 3 [OS=Homo sapiens]	2

Immunoglobulin lambda variable 3-21 [OS=Homo sapiens]	2
CD9 antigen [OS=Homo sapiens]	2
COP9 signalosome complex subunit 1 [OS=Homo sapiens]	2
Immunoglobulin lambda variable 3-25 [OS=Homo sapiens]	2
Isoform 2 of Septin-5 [OS=Homo sapiens]	2
Exportin-2 [OS=Homo sapiens]	2
Immunoglobulin lambda variable 7-46 [OS=Homo sapiens]	2
Apolipoprotein A-II [OS=Homo sapiens]	2
actin-related protein 3 [OS=Homo sapiens]	2
ATP synthase subunit alpha, mitochondrial [OS=Homo sapiens]	2
Angiotensinogen [OS=Homo sapiens]	2
ATP-dependent 6-phosphofructokinase, platelet type [OS=Homo sapiens]	2
Contactin-associated protein-like 2 [OS=Homo sapiens]	2
Insulin-like growth factor-binding protein complex acid labile subunit [OS=Homo sapiens]	2
Disks large homolog 1 [OS=Homo sapiens]	2
Interleukin-36 gamma [OS=Homo sapiens]	2
Apolipoprotein C-III [OS=Homo sapiens]	2
F-actin-capping protein subunit alpha-2 [OS=Homo sapiens]	2
GMP reductase 1 [OS=Homo sapiens]	2
ATP-citrate synthase [OS=Homo sapiens]	2

CD81 antigen [OS=Homo sapiens]	2
Guanine nucleotide-binding protein G(O) subunit alpha [OS=Homo sapiens]	2
E3 ubiquitin-protein ligase CHIP [OS=Homo sapiens]	2
Glutathione S-transferase Mu 1 [OS=Homo sapiens]	2
aflatoxin B1 aldehyde reductase member 2 [OS=Homo sapiens]	2
Glutathione S-transferase Mu 5 [OS=Homo sapiens]	2
Destrin [OS=Homo sapiens]	2
ATP-dependent RNA helicase DDX1 [OS=Homo sapiens]	2
Immunoglobulin kappa variable 2D-40 [OS=Homo sapiens]	2
Cell division control protein 42 homolog [OS=Homo sapiens]	2
Dihydrolipoyl dehydrogenase, mitochondrial [OS=Homo sapiens]	2
annexin A1 [OS=Homo sapiens]	2
heat shock 70 kDa protein 1A [OS=Homo sapiens]	2
Alpha-adducin [OS=Homo sapiens]	2
alpha-endosulfine [OS=Homo sapiens]	2
Glyceraldehyde-3-phosphate dehydrogenase, testis-specific [OS=Homo sapiens]	2
Eukaryotic translation initiation factor 5A-2 [OS=Homo sapiens]	2
amphiphysin [OS=Homo sapiens]	2
Amyloid-like protein 2 [OS=Homo sapiens]	2
Immunoglobulin lambda variable 2-8 [OS=Homo sapiens]	2

activator of 90 kDa heat shock protein ATPase homolog 1 [OS=Homo sapiens]	2
Heat shock protein beta-1 [OS=Homo sapiens]	2
Proteasome subunit alpha type-2 [OS=Homo sapiens]	2
Stathmin [OS=Homo sapiens]	2
Protein disulfide-isomerase A3 [OS=Homo sapiens]	2
T-complex protein 1 subunit beta [OS=Homo sapiens]	2
Protein NDRG2 [OS=Homo sapiens]	2
Neutral alpha-glucosidase AB [OS=Homo sapiens]	2
Neuronal-specific septin-3 [OS=Homo sapiens]	2
syntaxin-1A [OS=Homo sapiens]	2
syndecan-2 [OS=Homo sapiens]	2
Proteasome subunit alpha type-5 [OS=Homo sapiens]	2
Protein RUFY3 [OS=Homo sapiens]	2
UMP-CMP kinase [OS=Homo sapiens]	2
Vacuolar protein sorting-associated protein 35 [OS=Homo sapiens]	2
Protein S100-A12 [OS=Homo sapiens]	2
Synaptojanin-1 [OS=Homo sapiens]	2
protein S100-A14 [OS=Homo sapiens]	2
Vesicle-associated membrane protein 2 [OS=Homo sapiens]	2
Protein S100-A9 [OS=Homo sapiens]	2
Protein transport protein Sec31A [OS=Homo sapiens]	2
Neurofascin [OS=Homo sapiens]	2

Vesicular integral-membrane protein VIP36 [OS=Homo sapiens]	2
Phytanoyl-CoA hydroxylase-interacting protein-like [OS=Homo sapiens]	2
Ubiquitin thioesterase otub1 [OS=Homo sapiens]	2
Plasma serine protease inhibitor [OS=Homo sapiens]	2
Translin [OS=Homo sapiens]	2
Plastin-3 [OS=Homo sapiens]	2
Platelet-activating factor acetylhydrolase IB subunit alpha [OS=Homo sapiens]	2
plectin [OS=Homo sapiens]	2
phosphoacetylglucosamine mutase [OS=Homo sapiens]	2
Phosphatidylinositol-glycan-specific phospholipase D [OS=Homo sapiens]	2
Phosphatidylinositol-binding clathrin assembly protein [OS=Homo sapiens]	2
proteasome subunit beta type-5 [OS=Homo sapiens]	2
Programmed cell death 6-interacting protein [OS=Homo sapiens]	2
UTP--glucose-1-phosphate uridylyltransferase [OS=Homo sapiens]	2
Ubiquitin-60S ribosomal protein L40 [OS=Homo sapiens]	2
ProSAAS [OS=Homo sapiens]	2
Ubiquitin-conjugating enzyme E2 variant 2 [OS=Homo sapiens]	2
prostaglandin F2 receptor negative regulator [OS=Homo sapiens]	2
Peroxiredoxin-5, mitochondrial [OS=Homo sapiens]	2
Ubiquitin-fold modifier 1 [OS=Homo sapiens]	2

Peroxiredoxin-2 [OS=Homo sapiens]	2
UDP-N-acetylglucosamine--peptide N-acetylglucosaminyltransferase 110 kDa subunit [OS=Homo sapiens]	2
Peptidyl-prolyl cis-trans isomerase FKBP4 [OS=Homo sapiens]	2
Tyrosine--tRNA ligase, cytoplasmic [OS=Homo sapiens]	2
Serine protease HTRA1 [OS=Homo sapiens]	2
Ras-related protein Rab-14 [OS=Homo sapiens]	2
Nck-associated protein 1 [OS=Homo sapiens]	2
N(G),N(G)-dimethylarginine dimethylaminohydrolase 1 [OS=Homo sapiens]	2
Rap1 GTPase-GDP dissociation stimulator 1 [OS=Homo sapiens]	2
Rabphilin-3A [OS=Homo sapiens]	2
Serpin B4 [OS=Homo sapiens]	2
Ras-related protein Rab-6B [OS=Homo sapiens]	2
Ras-related protein Rab-2A [OS=Homo sapiens]	2
sorting nexin-5 [OS=Homo sapiens]	2
Serine/threonine-protein kinase PAK 3 [OS=Homo sapiens]	2
N-acetylmuramoyl-L-alanine amidase [OS=Homo sapiens]	2
Putative heat shock 70 kDa protein 7 [OS=Homo sapiens]	2
N(G),N(G)-dimethylarginine dimethylaminohydrolase 2 [OS=Homo sapiens]	2
Seizure protein 6 homolog [OS=Homo sapiens]	2
Neural cell adhesion molecule 2 [OS=Homo sapiens]	2

Putative protein FAM10A4 [OS=Homo sapiens]	2
Septin-8 [OS=Homo sapiens]	2
Dermcidin [OS=Homo sapiens]	1.857142857
Ficolin-3 [OS=Homo sapiens]	1.857142857
apolipoprotein M [OS=Homo sapiens]	1.833333333
Immunoglobulin lambda variable 2-11 [OS=Homo sapiens]	1.833333333
Complement component C8 beta chain [OS=Homo sapiens]	1.833333333
Proteasome subunit beta type-6 [OS=Homo sapiens]	1.8
Protein S100-A7 [OS=Homo sapiens]	1.8
Proteasome subunit beta type-7 [OS=Homo sapiens]	1.8
Adenosylhomocysteinase [OS=Homo sapiens]	1.75
Proteasome subunit alpha type-6 [OS=Homo sapiens]	1.75
F-box only protein 50 [OS=Homo sapiens]	1.75
peptidyl-prolyl cis-trans isomerase A [OS=Homo sapiens]	1.75
immunoglobulin kappa variable 1-8 [OS=Homo sapiens]	1.75
Proteasome subunit beta type-4 [OS=Homo sapiens]	1.75
immunoglobulin kappa variable 2-24 [OS=Homo sapiens]	1.75
Heat shock 70 kDa protein 4 [OS=Homo sapiens]	1.75
4-trimethylaminobutyraldehyde dehydrogenase [OS=Homo sapiens]	1.75
Immunoglobulin heavy variable 1-2 [OS=Homo sapiens]	1.714285714
Immunoglobulin lambda variable 3-19 [OS=Homo sapiens]	1.714285714

Testican-2 [OS=Homo sapiens]	1.666666667
Nucleoside diphosphate kinase A [OS=Homo sapiens]	1.666666667
immunoglobulin heavy variable 6-1 [OS=Homo sapiens]	1.666666667
Basement membrane-specific heparan sulfate proteoglycan core protein [OS=Homo sapiens]	1.666666667
glucose-6-phosphate isomerase [OS=Homo sapiens]	1.666666667
Ras-related C3 botulinum toxin substrate 3 [OS=Homo sapiens]	1.666666667
Endophilin-A2 [OS=Homo sapiens]	1.666666667
Complement C1q subcomponent subunit A [OS=Homo sapiens]	1.666666667
Protein S100-A8 [OS=Homo sapiens]	1.6
Properdin [OS=Homo sapiens]	1.6
gamma-glutamylcyclotransferase [OS=Homo sapiens]	1.6
Proteasome subunit alpha type-3 [OS=Homo sapiens]	1.6
Serum amyloid A-4 protein [OS=Homo sapiens]	1.571428571
immunoglobulin kappa variable 4-1 [OS=Homo sapiens]	1.571428571
Creatine kinase S-type, mitochondrial [OS=Homo sapiens]	1.5
Heat shock protein 105 kDa [OS=Homo sapiens]	1.5
Profilin-2 [OS=Homo sapiens]	1.5
Cathepsin B [OS=Homo sapiens]	1.5
Plexin domain-containing protein 2 [OS=Homo sapiens]	1.5
Glutathione peroxidase 3 [OS=Homo sapiens]	1.5
CD63 antigen [OS=Homo sapiens]	1.5
coactosin-like protein [OS=Homo sapiens]	1.5

immunoglobulin lambda variable 3-27 [OS=Homo sapiens]	1.5
Septin-11 [OS=Homo sapiens]	1.5
Platelet factor 4 [OS=Homo sapiens]	1.5
Platelet basic protein [OS=Homo sapiens]	1.5
Succinyl-CoA:3-ketoacid coenzyme A transferase 1, mitochondrial [OS=Homo sapiens]	1.5
GTP-binding nuclear protein RAN [OS=Homo sapiens]	1.5
citrate synthase, mitochondrial [OS=Homo sapiens]	1.5
Cysteine-rich secretory protein 3 [OS=Homo sapiens]	1.5
Serum paraoxonase/lactonase 3 [OS=Homo sapiens]	1.5
Immunoglobulin heavy constant delta [OS=Homo sapiens]	1.5
Calcineurin subunit B type 1 [OS=Homo sapiens]	1.5
Calcium-dependent secretion activator 1 [OS=Homo sapiens]	1.5
Fatty acid synthase [OS=Homo sapiens]	1.5
clathrin light chain A [OS=Homo sapiens]	1.5
Ferritin heavy chain [OS=Homo sapiens]	1.5
Carboxypeptidase A4 [OS=Homo sapiens]	1.5
Histone H2A type 2-C [OS=Homo sapiens]	1.5
Calreticulin [OS=Homo sapiens]	1.5
Ribonuclease 4 [OS=Homo sapiens]	1.5
Seizure 6-like protein [OS=Homo sapiens]	1.5
Serine protease inhibitor Kazal-type 5 [OS=Homo sapiens]	1.5

protein S100-A11 [OS=Homo sapiens]	1.5
Complement component C8 gamma chain [OS=Homo sapiens]	1.5
immunoglobulin heavy variable 3-64D [OS=Homo sapiens]	1.5
Serine/threonine-protein phosphatase 2A activator [OS=Homo sapiens]	1.5
Hepatocyte growth factor-like protein [OS=Homo sapiens]	1.5
Prostaglandin E synthase 3 [OS=Homo sapiens]	1.5
Prosaposin [OS=Homo sapiens]	1.5
Brain-specific angiogenesis inhibitor 1-associated protein 2 [OS=Homo sapiens]	1.5
corneodesmosin [OS=Homo sapiens]	1.5
proteasome subunit beta type-3 [OS=Homo sapiens]	1.5
F-actin-capping protein subunit beta [OS=Homo sapiens]	1.5
Peroxiredoxin-6 [OS=Homo sapiens]	1.5
Neutrophil defensin 1 [OS=Homo sapiens]	1.5
Neuropeptide-like protein C4orf48 [OS=Homo sapiens]	1.5
Lysosome-associated membrane glycoprotein 1 [OS=Homo sapiens]	1.5
Vinculin [OS=Homo sapiens]	1.5
microtubule-associated protein 1B [OS=Homo sapiens]	1.5
loricrin [OS=Homo sapiens]	1.5
Neuroblast differentiation-associated protein AHNAK [OS=Homo sapiens]	1.5
alcohol dehydrogenase [NADP(+)] [OS=Homo sapiens]	1.5

adenylyl cyclase-associated protein 1 [OS=Homo sapiens]	1.5
10 kDa heat shock protein, mitochondrial [OS=Homo sapiens]	1.5
C-reactive protein [OS=Homo sapiens]	1.428571429
Leucine-rich alpha-2-glycoprotein [OS=Homo sapiens]	1.4
immunoglobulin heavy variable 2-70D [OS=Homo sapiens]	1.4
Prolactin-inducible protein [OS=Homo sapiens]	1.4
Beta-2-microglobulin [OS=Homo sapiens]	1.4
EGF-containing fibulin-like extracellular matrix protein 1 [OS=Homo sapiens]	1.4
Insulin-like growth factor-binding protein 7 [OS=Homo sapiens]	1.333333333
Arachidonate 12-lipoxygenase, 12R-type [OS=Homo sapiens]	1.333333333
Transcriptional activator protein Pur-alpha [OS=Homo sapiens]	1.333333333
Pigment epithelium-derived factor [OS=Homo sapiens]	1.333333333
myelin basic protein [OS=Homo sapiens]	1.333333333
corticosteroid-binding globulin [OS=Homo sapiens]	1.333333333
cathelicidin antimicrobial peptide [OS=Homo sapiens]	1.333333333
Polymeric immunoglobulin receptor [OS=Homo sapiens]	1.333333333
MARCKS-related protein [OS=Homo sapiens]	1.333333333
gasdermin-A [OS=Homo sapiens]	1.333333333
Nucleophosmin [OS=Homo sapiens]	1.333333333
Antileukoproteinase [OS=Homo sapiens]	1.333333333

complexin-2 [OS=Homo sapiens]	1.333333333
Filamin-A [OS=Homo sapiens]	1.333333333
Astrocytic phosphoprotein PEA-15 [OS=Homo sapiens]	1.333333333
thioredoxin [OS=Homo sapiens]	1.285714286
Ganglioside GM2 activator [OS=Homo sapiens]	1.25
phosphatidylethanolamine-binding protein 1 [OS=Homo sapiens]	1.25
Histone H2B type 2-E [OS=Homo sapiens]	1.25
Cornifin-B [OS=Homo sapiens]	1.25
Fascin [OS=Homo sapiens]	1.25
Transketolase [OS=Homo sapiens]	1.25
Cystatin-M [OS=Homo sapiens]	1.25
Tetranectin [OS=Homo sapiens]	1.2
small proline-rich protein 2D [OS=Homo sapiens]	1.2
immunoglobulin lambda variable 1-51 [OS=Homo sapiens]	1.2
Guanine nucleotide-binding protein subunit beta-4 [OS=Homo sapiens]	1.2
phospholipid transfer protein [OS=Homo sapiens]	1.166666667
Immunoglobulin lambda variable 1-47 [OS=Homo sapiens]	1.142857143
Collagen alpha-1(VI) chain [OS=Homo sapiens]	1
Sorting nexin-1 [OS=Homo sapiens]	1
small ubiquitin-related modifier 4 [OS=Homo sapiens]	1
Visinin-like protein 1 [OS=Homo sapiens]	1
Collagen alpha-3(VI) chain [OS=Homo sapiens]	1

Coiled-coil domain-containing protein 57 [OS=Homo sapiens]	1
S-methylmethionine--homocysteine S-methyltransferase BHMT2 [OS=Homo sapiens]	1
40S ribosomal protein SA [OS=Homo sapiens]	1
Solute carrier family 12 member 5 [OS=Homo sapiens]	1
Collagen alpha-1(III) chain [OS=Homo sapiens]	1
40S ribosomal protein S9 [OS=Homo sapiens]	1
Spermatogenesis-associated protein 31C1 [OS=Homo sapiens]	1
Collagen alpha-1(I) chain [OS=Homo sapiens]	1
Coiled-coil domain-containing protein 81 [OS=Homo sapiens]	1
60S ribosomal protein L34 [OS=Homo sapiens]	1
Chromodomain-helicase-DNA-binding protein 3 [OS=Homo sapiens]	1
Chondroitin sulfate proteoglycan 5 [OS=Homo sapiens]	1
Chondroadherin [OS=Homo sapiens]	1
Succinate--CoA ligase [GDP-forming] subunit beta, mitochondrial [OS=Homo sapiens]	1
Succinate-semialdehyde dehydrogenase, mitochondrial [OS=Homo sapiens]	1
Spectrin alpha chain, non-erythrocytic 1 [OS=Homo sapiens]	1
Charged multivesicular body protein 4b [OS=Homo sapiens]	1
60S ribosomal protein L3 [OS=Homo sapiens]	1
Charged multivesicular body protein 3 [OS=Homo sapiens]	1
60S ribosomal protein L36a [OS=Homo sapiens]	1

SUMO-conjugating enzyme ubc9 [OS=Homo sapiens]	1
Superoxide dismutase [Cu-Zn] [OS=Homo sapiens]	1
Synaptic vesicle membrane protein VAT-1 homolog [OS=Homo sapiens]	1
abl interactor 1 [OS=Homo sapiens]	1
Chloride intracellular channel protein 4 [OS=Homo sapiens]	1
s-phase kinase-associated protein 1 [OS=Homo sapiens]	1
Coatomer subunit delta [OS=Homo sapiens]	1
60S ribosomal protein L12 [OS=Homo sapiens]	1
Coatomer subunit beta' [OS=Homo sapiens]	1
Sorting nexin-27 [OS=Homo sapiens]	1
60S ribosomal protein L13 [OS=Homo sapiens]	1
Spatacsin [OS=Homo sapiens]	1
Cilia- and flagella-associated protein 74 [OS=Homo sapiens]	1
SH3 domain-binding glutamic acid-rich-like protein 3 [OS=Homo sapiens]	1
Succinate--CoA ligase [ADP-forming] subunit beta, mitochondrial [OS=Homo sapiens]	1
Coagulation factor IX [OS=Homo sapiens]	1
60S ribosomal protein L23 [OS=Homo sapiens]	1
Spondin-1 [OS=Homo sapiens]	1
Src substrate cortactin [OS=Homo sapiens]	1
60S ribosomal protein L26 [OS=Homo sapiens]	1
Sorting nexin-2 [OS=Homo sapiens]	1

60S ribosomal protein L17 [OS=Homo sapiens]	1
Septin-9 [OS=Homo sapiens]	1
COP9 signalosome complex subunit 3 [OS=Homo sapiens]	1
COP9 signalosome complex subunit 4 [OS=Homo sapiens]	1
COP9 signalosome complex subunit 5 [OS=Homo sapiens]	1
Copine-6 [OS=Homo sapiens]	1
WW domain-binding protein 2 [OS=Homo sapiens]	1
Serine protease inhibitor Kazal-type 7 [OS=Homo sapiens]	1
Coronin-1B [OS=Homo sapiens]	1
xaa-Pro aminopeptidase 1 [OS=Homo sapiens]	1
xaa-Pro dipeptidase [OS=Homo sapiens]	1
Coronin-7 [OS=Homo sapiens]	1
X-ray repair cross-complementing protein 5 [OS=Homo sapiens]	1
Xylosyltransferase 1 [OS=Homo sapiens]	1
SH3-containing GRB2-like protein 3-interacting protein 1 [OS=Homo sapiens]	1
Cullin-3 [OS=Homo sapiens]	1
WAS/WASL-interacting protein family member 3 [OS=Homo sapiens]	1
zinc finger protein 26 [OS=Homo sapiens]	1
Cystatin-C [OS=Homo sapiens]	1
Cysteine and glycine-rich protein 1 [OS=Homo sapiens]	1
Zinc finger protein 350 [OS=Homo sapiens]	1

cytochrome c [OS=Homo sapiens]	1
Cytochrome c oxidase subunit 6B1 [OS=Homo sapiens]	1
Cytoplasmic aconitate hydratase [OS=Homo sapiens]	1
Septin-5 [OS=Homo sapiens]	1
cytosol aminopeptidase [OS=Homo sapiens]	1
Septin-14 [OS=Homo sapiens]	1
D-3-phosphoglycerate dehydrogenase [OS=Homo sapiens]	1
DCC-interacting protein 13-alpha [OS=Homo sapiens]	1
1,4-alpha-glucan-branching enzyme [OS=Homo sapiens]	1
C-terminal-binding protein 1 [OS=Homo sapiens]	1
SH3 domain-binding glutamic acid-rich-like protein [OS=Homo sapiens]	1
Complement C1q tumor necrosis factor-related protein 3 [OS=Homo sapiens]	1
Vitamin K-dependent protein Z [OS=Homo sapiens]	1
Small nuclear ribonucleoprotein Sm D2 [OS=Homo sapiens]	1
Small glutamine-rich tetratricopeptide repeat-containing protein alpha [OS=Homo sapiens]	1
40S ribosomal protein S3 [OS=Homo sapiens]	1
VPS10 domain-containing receptor SorCS1 [OS=Homo sapiens]	1
Sialic acid-binding Ig-like lectin 16 [OS=Homo sapiens]	1
sialic acid synthase [OS=Homo sapiens]	1
Shootin-1 [OS=Homo sapiens]	1
V-type proton ATPase subunit C 1 [OS=Homo sapiens]	1

2-oxoglutarate dehydrogenase-like, mitochondrial [OS=Homo sapiens]	1
2-hydroxyacylsphingosine 1-beta-galactosyltransferase [OS=Homo sapiens]	1
26S proteasome regulatory subunit 8 [OS=Homo sapiens]	1
Serine/arginine-rich splicing factor 3 [OS=Homo sapiens]	1
Complement factor D [OS=Homo sapiens]	1
40S ribosomal protein S5 [OS=Homo sapiens]	1
1-phosphatidylinositol 4,5-bisphosphate phosphodiesterase eta-1 [OS=Homo sapiens]	1
Serine/threonine-protein kinase/endoribonuclease IRE1 [OS=Homo sapiens]	1
WAP four-disulfide core domain protein 12 [OS=Homo sapiens]	1
Serine/threonine-protein phosphatase 5 [OS=Homo sapiens]	1
V-type proton ATPase subunit G 1 [OS=Homo sapiens]	1
V-type proton ATPase subunit G 2 [OS=Homo sapiens]	1
Complement component 1 Q subcomponent-binding protein, mitochondrial [OS=Homo sapiens]	1
Serpin B7 [OS=Homo sapiens]	1
Serum amyloid A-2 protein [OS=Homo sapiens]	1
26S proteasome non-ATPase regulatory subunit 3 [OS=Homo sapiens]	1
26S proteasome regulatory subunit 10B [OS=Homo sapiens]	1
26S proteasome regulatory subunit 6B [OS=Homo sapiens]	1
Serine/threonine-protein kinase Nek11 [OS=Homo sapiens]	1

Serine-threonine kinase receptor-associated protein [OS=Homo sapiens]	1
TIP41-like protein [OS=Homo sapiens]	1
Vascular cell adhesion protein 1 [OS=Homo sapiens]	1
Thioredoxin-dependent peroxide reductase, mitochondrial [OS=Homo sapiens]	1
ATP-dependent RNA helicase DHX36 [OS=Homo sapiens]	1
U6 snRNA-associated Sm-like protein LSm8 [OS=Homo sapiens]	1
U1 SMALL NUCLEAR RIBONUCLEOPROTEIN A [OS=Homo sapiens]	1
Alpha-centractin [OS=Homo sapiens]	1
ATP-dependent RNA helicase DDX55 [OS=Homo sapiens]	1
Basic leucine zipper and W2 domain-containing protein 1 [OS=Homo sapiens]	1
Tissue factor pathway inhibitor [OS=Homo sapiens]	1
Titin [OS=Homo sapiens]	1
Toll-interacting protein [OS=Homo sapiens]	1
ATPase WRNIP1 [OS=Homo sapiens]	1
Trafficking protein particle complex subunit 3 [OS=Homo sapiens]	1
U1 small nuclear ribonucleoprotein 70 kDa [OS=Homo sapiens]	1
ATP-dependent RNA helicase Dhx29 [OS=Homo sapiens]	1
Alpha-2,8-sialyltransferase 8B [OS=Homo sapiens]	1
Beta-centractin [OS=Homo sapiens]	1
alcohol dehydrogenase class-3 [OS=Homo sapiens]	1

Aldehyde dehydrogenase, mitochondrial [OS=Homo sapiens]	1
aldose reductase [OS=Homo sapiens]	1
Ras-related protein Ral-A [OS=Homo sapiens]	1
band 4.1-like protein 2 [OS=Homo sapiens]	1
Ubiquitin-conjugating enzyme E2 Z [OS=Homo sapiens]	1
Transcription initiation factor TFIID subunit 2 [OS=Homo sapiens]	1
Ubiquitin-conjugating enzyme E2 variant 1 [OS=Homo sapiens]	1
ubiquitin-conjugating enzyme E2 N [OS=Homo sapiens]	1
Ubiquitin carboxyl-terminal hydrolase 14 [OS=Homo sapiens]	1
Alpha-aminoadipic semialdehyde dehydrogenase [OS=Homo sapiens]	1
alpha-amylase 1 [OS=Homo sapiens]	1
beta-1,4-glucuronyltransferase 1 [OS=Homo sapiens]	1
Beta-adducin [OS=Homo sapiens]	1
tubulin-folding cofactor B [OS=Homo sapiens]	1
Translation initiation factor eIF-2B subunit beta [OS=Homo sapiens]	1
annexin A11 [OS=Homo sapiens]	1
Transmembrane emp24 domain-containing protein 4 [OS=Homo sapiens]	1
Apolipoprotein A-V [OS=Homo sapiens]	1
Tripeptidyl-peptidase 2 [OS=Homo sapiens]	1
Transaldolase [OS=Homo sapiens]	1
Tropomyosin alpha-4 chain [OS=Homo sapiens]	1

transgelin [OS=Homo sapiens]	1
AP2-associated protein kinase 1 [OS=Homo sapiens]	1
TSC22 domain family protein 3 [OS=Homo sapiens]	1
tubulin alpha-3C/D chain [OS=Homo sapiens]	1
Tubulin polymerization-promoting protein [OS=Homo sapiens]	1
Annexin A7 [OS=Homo sapiens]	1
annexin A3 [OS=Homo sapiens]	1
Tropomodulin-2 [OS=Homo sapiens]	1
Tumor protein D54 [OS=Homo sapiens]	1
Tetraspanin-6 [OS=Homo sapiens]	1
Transcription intermediary factor 1-beta [OS=Homo sapiens]	1
AMP deaminase 2 [OS=Homo sapiens]	1
Arf-GAP domain and FG repeat-containing protein 1 [OS=Homo sapiens]	1
Tyrosine-protein phosphatase non-receptor type 4 [OS=Homo sapiens]	1
Tyrosine-protein kinase Tec [OS=Homo sapiens]	1
Tubulin-specific chaperone A [OS=Homo sapiens]	1
tumor susceptibility gene 101 protein [OS=Homo sapiens]	1
Apolipoprotein C-IV [OS=Homo sapiens]	1
Tubulin-specific chaperone cofactor E-like protein [OS=Homo sapiens]	1
Transcriptional adapter 2-beta [OS=Homo sapiens]	1
Apolipoprotein F [OS=Homo sapiens]	1

Transforming protein RhoA [OS=Homo sapiens]	1
Anillin [OS=Homo sapiens]	1
Transcription factor E2F8 [OS=Homo sapiens]	1
Amyloid-like protein 1 [OS=Homo sapiens]	1
Acylpyruvase FAHD1, mitochondrial [OS=Homo sapiens]	1
cartilage acidic protein 1 [OS=Homo sapiens]	1
UV excision repair protein RAD23 homolog B [OS=Homo sapiens]	1
Carnitine O-acetyltransferase [OS=Homo sapiens]	1
Carboxypeptidase n subunit 2 [OS=Homo sapiens]	1
acyl-protein thioesterase 2 [OS=Homo sapiens]	1
Adapter molecule crk [OS=Homo sapiens]	1
Synaptosomal-associated protein 25 [OS=Homo sapiens]	1
Synaptophysin [OS=Homo sapiens]	1
Carbonyl reductase [NADPH] 1 [OS=Homo sapiens]	1
CAP-Gly domain-containing linker protein 2 [OS=Homo sapiens]	1
cAMP-regulated phosphoprotein 21 [OS=Homo sapiens]	1
cAMP-dependent protein kinase type II-beta regulatory subunit [OS=Homo sapiens]	1
cAMP-dependent protein kinase catalytic subunit alpha [OS=Homo sapiens]	1
Alanine--tRNA ligase, cytoplasmic [OS=Homo sapiens]	1
Carboxypeptidase N catalytic chain [OS=Homo sapiens]	1

Vesicle-associated membrane protein-associated protein B/C [OS=Homo sapiens]	1
CD151 antigen [OS=Homo sapiens]	1
C-C motif chemokine 18 [OS=Homo sapiens]	1
Acetyl-CoA acetyltransferase, cytosolic [OS=Homo sapiens]	1
CB1 cannabinoid receptor-interacting protein 1 [OS=Homo sapiens]	1
Acetyl-CoA acetyltransferase, mitochondrial [OS=Homo sapiens]	1
caveolae-associated protein 1 [OS=Homo sapiens]	1
actin-related protein 2/3 complex subunit 5-like protein [OS=Homo sapiens]	1
Acidic leucine-rich nuclear phosphoprotein 32 family member B [OS=Homo sapiens]	1
Actin-related protein 2/3 complex subunit 5 [OS=Homo sapiens]	1
vesicle-associated membrane protein-associated protein A [OS=Homo sapiens]	1
Beta-arrestin-1 [OS=Homo sapiens]	1
Actin-related protein 2/3 complex subunit 1A [OS=Homo sapiens]	1
annexin A5 [OS=Homo sapiens]	1
Actin-related protein 2/3 complex subunit 3 [OS=Homo sapiens]	1
adaptin ear-binding coat-associated protein 1 [OS=Homo sapiens]	1
Synaptogyrin-1 [OS=Homo sapiens]	1
Testis-specific serine/threonine-protein kinase 4 [OS=Homo sapiens]	1
BRO1 domain-containing protein BROX [OS=Homo sapiens]	1

T-complex protein 1 subunit eta [OS=Homo sapiens]	1
Branched-chain-amino-acid aminotransferase, cytosolic [OS=Homo sapiens]	1
Adhesion G protein-coupled receptor L2 [OS=Homo sapiens]	1
Adipocyte plasma membrane-associated protein [OS=Homo sapiens]	1
syndecan-1 [OS=Homo sapiens]	1
Tenascin-R [OS=Homo sapiens]	1
T-complex protein 1 subunit alpha [OS=Homo sapiens]	1
UPF0160 protein MYG1, mitochondrial [OS=Homo sapiens]	1
Uncharacterized protein KIAA0513 [OS=Homo sapiens]	1
Uncharacterized protein C6orf62 [OS=Homo sapiens]	1
Uncharacterized protein C18orf63 [OS=Homo sapiens]	1
A-kinase anchor protein 9 [OS=Homo sapiens]	1
Synaptobrevin homolog YKT6 [OS=Homo sapiens]	1
Urokinase-type plasminogen activator [OS=Homo sapiens]	1
TBC1 domain family member 4 [OS=Homo sapiens]	1
Calpain-2 catalytic subunit [OS=Homo sapiens]	1
Calpain-1 catalytic subunit [OS=Homo sapiens]	1
Calpain small subunit 1 [OS=Homo sapiens]	1
Calnexin [OS=Homo sapiens]	1
Syntenin-1 [OS=Homo sapiens]	1
Adenylate kinase 4, mitochondrial [OS=Homo sapiens]	1

T-complex protein 1 subunit delta [OS=Homo sapiens]	1
Talin-2 [OS=Homo sapiens]	1
BTB/POZ domain-containing protein KCTD12 [OS=Homo sapiens]	1
Adenylate kinase isoenzyme 1 [OS=Homo sapiens]	1
Calcium/calmodulin-dependent 3',5'-cyclic nucleotide phosphodiesterase 1B [OS=Homo sapiens]	1
adenylosuccinate lyase [OS=Homo sapiens]	1
T-cell activation Rho GTPase-activating protein [OS=Homo sapiens]	1
Cadherin-1 [OS=Homo sapiens]	1
thimet oligopeptidase [OS=Homo sapiens]	1
calmodulin regulator protein PCP4 [OS=Homo sapiens]	1
phytanoyl-CoA hydroxylase-interacting protein [OS=Homo sapiens]	1
proteasome subunit beta type-2 [OS=Homo sapiens]	1
Phosphoglucomutase-2 [OS=Homo sapiens]	1
Phosphoglycerate kinase 2 [OS=Homo sapiens]	1
Isoform 2 of Cilia- and flagella-associated protein 44 [OS=Homo sapiens]	1
Phosphoribosylformylglycinamidine synthase [OS=Homo sapiens]	1
Isoform 1 of Pannexin-2 [OS=Homo sapiens]	1
Isoform 1 of BRISC and BRCA1-A complex member 2 [OS=Homo sapiens]	1
Phosphofurin acidic cluster sorting protein 1 [OS=Homo sapiens]	1
isocitrate dehydrogenase [NAD] subunit beta, mitochondrial [OS=Homo sapiens]	1

Isoform 2 of Protocadherin gamma-C5 [OS=Homo sapiens]	1
(E3-independent) E2 ubiquitin-conjugating enzyme [OS=Homo sapiens]	1
Interleukin enhancer-binding factor 3 [OS=Homo sapiens]	1
Interleukin enhancer-binding factor 2 [OS=Homo sapiens]	1
PITH domain-containing protein 1 [OS=Homo sapiens]	1
Plasma membrane calcium-transporting ATPase 2 [OS=Homo sapiens]	1
Plasma membrane calcium-transporting ATPase 4 [OS=Homo sapiens]	1
Insulin-like growth factor-binding protein 5 [OS=Homo sapiens]	1
phosphoserine aminotransferase [OS=Homo sapiens]	1
Isoform 5 of Tumor necrosis factor receptor superfamily member 1A [OS=Homo sapiens]	1
Peroxiredoxin-4 [OS=Homo sapiens]	1
peroxisomal multifunctional enzyme type 2 [OS=Homo sapiens]	1
Peroxisome proliferator-activated receptor delta [OS=Homo sapiens]	1
Kallistatin [OS=Homo sapiens]	1
PH and SEC7 domain-containing protein 3 [OS=Homo sapiens]	1
IST1 homolog [OS=Homo sapiens]	1
PHD finger protein 24 [OS=Homo sapiens]	1
Isoform 2 of Glyoxalase domain-containing protein 4 [OS=Homo sapiens]	1
phosphatidylcholine-sterol acyltransferase [OS=Homo sapiens]	1

Insulin-like growth factor II [OS=Homo sapiens]	1
Isoform 4 of Serine/threonine-protein phosphatase 2A 56 kDa regulatory subunit gamma isoform [OS=Homo sapiens]	1
Isoform 3 of Zinc finger protein 568 [OS=Homo sapiens]	1
Isoform 3 of PDZ domain-containing protein 7 [OS=Homo sapiens]	1
Isoform 3 of LIM and calponin homology domains-containing protein 1 [OS=Homo sapiens]	1
Isoform 2 of V-type proton ATPase 116 kDa subunit a isoform 1 [OS=Homo sapiens]	1
Phosphatidylinositol transfer protein alpha isoform [OS=Homo sapiens]	1
Isoform 2 of Transmembrane protease serine 6 [OS=Homo sapiens]	1
Isoform 7 of Inositol hexakisphosphate and diphosphoinositol-pentakisphosphate kinase 1 [OS=Homo sapiens]	1
immunoglobulin kappa variable 1-27 [OS=Homo sapiens]	1
Immunoglobulin kappa variable 3D-15 [OS=Homo sapiens]	1
immunoglobulin kappa variable 3D-11 [OS=Homo sapiens]	1
Probable carboxypeptidase X1 [OS=Homo sapiens]	1
Immunoglobulin kappa variable 3-15 [OS=Homo sapiens]	1
Probable sodium-coupled neutral amino acid transporter 6 [OS=Homo sapiens]	1
Probable Xaa-Pro aminopeptidase 3 [OS=Homo sapiens]	1
Pro-cathepsin H [OS=Homo sapiens]	1

insulin-like growth factor-binding protein 4 [OS=Homo sapiens]	1
Immunoglobulin kappa variable 1-33 [OS=Homo sapiens]	1
Immunoglobulin kappa variable 6D-21 [OS=Homo sapiens]	1
Immunoglobulin kappa variable 1-17 [OS=Homo sapiens]	1
Procollagen C-endopeptidase enhancer 2 [OS=Homo sapiens]	1
Procollagen-lysine,2-oxoglutarate 5-dioxygenase 1 [OS=Homo sapiens]	1
profilin-1 [OS=Homo sapiens]	1
prolyl endopeptidase [OS=Homo sapiens]	1
Prostaglandin-H2 D-isomerase [OS=Homo sapiens]	1
Ras-related protein Rap-1b [OS=Homo sapiens]	1
immunoglobulin kappa variable 1-6 [OS=Homo sapiens]	1
immunoglobulin lambda variable 4-69 [OS=Homo sapiens]	1
Peptidyl-prolyl cis-trans isomerase FKBP1A [OS=Homo sapiens]	1
Insulin [OS=Homo sapiens]	1
Inositol 1,4,5-trisphosphate receptor type 1 [OS=Homo sapiens]	1
Importin subunit beta-1 [OS=Homo sapiens]	1
Plasminogen activator inhibitor 2 [OS=Homo sapiens]	1
immunoglobulin lambda variable 9-49 [OS=Homo sapiens]	1
immunoglobulin lambda variable 8-61 [OS=Homo sapiens]	1

Prefoldin subunit 6 [OS=Homo sapiens]	1
Immunoglobulin lambda variable 5-39 [OS=Homo sapiens]	1
Immunoglobulin kappa variable 6-21 [OS=Homo sapiens]	1
Platelet-activating factor acetylhydrolase IB subunit gamma [OS=Homo sapiens]	1
immunoglobulin lambda variable 3-16 [OS=Homo sapiens]	1
Pleiotrophin [OS=Homo sapiens]	1
immunoglobulin lambda variable 2-18 [OS=Homo sapiens]	1
Immunoglobulin lambda variable 1-40 [OS=Homo sapiens]	1
immunoglobulin lambda variable 10-54 [OS=Homo sapiens]	1
Plexin-A3 [OS=Homo sapiens]	1
Plasminogen activator inhibitor 1 RNA-binding protein [OS=Homo sapiens]	1
immunoglobulin lambda variable 6-57 [OS=Homo sapiens]	1
Neprilysin [OS=Homo sapiens]	1
Mannosyl-oligosaccharide 1,2-alpha-mannosidase IA [OS=Homo sapiens]	1
Mannose-binding protein C [OS=Homo sapiens]	1
Mannan-binding lectin serine protease 2 [OS=Homo sapiens]	1
Mammaglobin-B [OS=Homo sapiens]	1
Mammaglobin-A [OS=Homo sapiens]	1
NEDD8 [OS=Homo sapiens]	1

NEDD8-activating enzyme E1 regulatory subunit [OS=Homo sapiens]	1
Neurocalcin-delta [OS=Homo sapiens]	1
MAGUK p55 subfamily member 2 [OS=Homo sapiens]	1
Metallothionein-1F [OS=Homo sapiens]	1
Neurabin-2 [OS=Homo sapiens]	1
lysosomal protective protein [OS=Homo sapiens]	1
Lymphocyte antigen 6H [OS=Homo sapiens]	1
Low-density lipoprotein receptor-related protein 2 [OS=Homo sapiens]	1
Low molecular weight phosphotyrosine protein phosphatase [OS=Homo sapiens]	1
Neural Wiskott-Aldrich syndrome protein [OS=Homo sapiens]	1
peroxidasin homolog [OS=Homo sapiens]	1
MAGUK p55 subfamily member 4 [OS=Homo sapiens]	1
Microtubule-associated proteins 1A/1B light chain 3A [OS=Homo sapiens]	1
Na(+)/H(+) exchange regulatory cofactor NHE-RF1 [OS=Homo sapiens]	1
N-acetyl-D-glucosamine kinase [OS=Homo sapiens]	1
NAD(P)H-hydrate epimerase [OS=Homo sapiens]	1
Mucin-like protein 1 [OS=Homo sapiens]	1
monoglyceride lipase [OS=Homo sapiens]	1
NAD-dependent malic enzyme, mitochondrial [OS=Homo sapiens]	1
Mitogen-activated protein kinase 9 [OS=Homo sapiens]	1
MAP7 domain-containing protein 2 [OS=Homo sapiens]	1

mitochondrial-processing peptidase subunit beta [OS=Homo sapiens]	1
Metalloproteinase inhibitor 2 [OS=Homo sapiens]	1
Microtubule-associated protein tau [OS=Homo sapiens]	1
Microtubule-associated protein RP/EB family member 2 [OS=Homo sapiens]	1
NADP-dependent malic enzyme [OS=Homo sapiens]	1
Microtubule-associated protein 1A [OS=Homo sapiens]	1
Methylmalonate-semialdehyde dehydrogenase [acylating], mitochondrial [OS=Homo sapiens]	1
Methionine--tRNA ligase, cytoplasmic [OS=Homo sapiens]	1
metallothionein-1X [OS=Homo sapiens]	1
LIM domain only protein 3 [OS=Homo sapiens]	1
NADH dehydrogenase [ubiquinone] flavoprotein 2, mitochondrial [OS=Homo sapiens]	1
OTU domain-containing protein 4 [OS=Homo sapiens]	1
Noelin-2 [OS=Homo sapiens]	1
NSFL1 cofactor p47 [OS=Homo sapiens]	1
nuclear mitotic apparatus protein 1 [OS=Homo sapiens]	1
nuclear transport factor 2 [OS=Homo sapiens]	1
Nuclear ubiquitous casein and cyclin-dependent kinase substrate 1 [OS=Homo sapiens]	1
Nuclease-sensitive element-binding protein 1 [OS=Homo sapiens]	1
Nucleolin [OS=Homo sapiens]	1
Liprin-alpha-3 [OS=Homo sapiens]	1

Ornithine aminotransferase, mitochondrial [OS=Homo sapiens]	1
Neuronal membrane glycoprotein M6-b [OS=Homo sapiens]	1
Oxysterol-binding protein 1 [OS=Homo sapiens]	1
Keratin, type II cuticular Hb2 [OS=Homo sapiens]	1
Paraspeckle component 1 [OS=Homo sapiens]	1
Patched domain-containing protein 3 [OS=Homo sapiens]	1
peptidyl-prolyl cis-trans isomerase B [OS=Homo sapiens]	1
peptidyl-prolyl cis-trans isomerase D [OS=Homo sapiens]	1
Immunoglobulin heavy variable 2-26 [OS=Homo sapiens]	1
Nucleosome assembly protein 1-like 4 [OS=Homo sapiens]	1
Neuroligin-2 [OS=Homo sapiens]	1
Leukocyte cell-derived chemotaxin-2 [OS=Homo sapiens]	1
leucine carboxyl methyltransferase 1 [OS=Homo sapiens]	1
Latexin [OS=Homo sapiens]	1
late cornified envelope protein 1F [OS=Homo sapiens]	1
Neurofilament light polypeptide [OS=Homo sapiens]	1
Laminin subunit beta-1 [OS=Homo sapiens]	1
lactoylglutathione lyase [OS=Homo sapiens]	1
Noelin [OS=Homo sapiens]	1
Lactadherin [OS=Homo sapiens]	1

neuronal pentraxin receptor [OS=Homo sapiens]	1
Kinesin-like protein KIF2A [OS=Homo sapiens]	1
Ketimine reductase mu-crystallin [OS=Homo sapiens]	1
Neuroligin-3 [OS=Homo sapiens]	1
Keratin-associated protein 3-3 [OS=Homo sapiens]	1
Keratin-associated protein 3-1 [OS=Homo sapiens]	1
Keratin-associated protein 2-4 [OS=Homo sapiens]	1
Neuroligin-4, X-linked [OS=Homo sapiens]	1
peptidyl-prolyl cis-trans isomerase FKBP3 [OS=Homo sapiens]	1
Neurofilament medium polypeptide [OS=Homo sapiens]	1
Receptor-type tyrosine-protein phosphatase F [OS=Homo sapiens]	1
Proteasome activator complex subunit 1 [OS=Homo sapiens]	1
Ras-related protein Rab-6A [OS=Homo sapiens]	1
Fetuin-B [OS=Homo sapiens]	1
FERM and PDZ domain-containing protein 3 [OS=Homo sapiens]	1
Fatty acid-binding protein, heart [OS=Homo sapiens]	1
Ezrin [OS=Homo sapiens]	1
Intron-binding protein aquarius [OS=Homo sapiens]	1
Fibrosin-1-like protein [OS=Homo sapiens]	1
Ras-related protein R-Ras2 [OS=Homo sapiens]	1
Ras-related protein Rab-11A [OS=Homo sapiens]	1

Eukaryotic translation initiation factor 5A-1-like [OS=Homo sapiens]	1
Eukaryotic translation initiation factor 4H [OS=Homo sapiens]	1
eukaryotic translation initiation factor 4B [OS=Homo sapiens]	1
Eukaryotic translation initiation factor 3 subunit I [OS=Homo sapiens]	1
Eukaryotic translation initiation factor 3 subunit B [OS=Homo sapiens]	1
Eukaryotic translation initiation factor 2 subunit 1 [OS=Homo sapiens]	1
Eukaryotic peptide chain release factor subunit 1 [OS=Homo sapiens]	1
Extracellular glycoprotein lacritin [OS=Homo sapiens]	1
Gamma-glutamyl hydrolase [OS=Homo sapiens]	1
glucosylceramidase [OS=Homo sapiens]	1
Glucosidase 2 subunit beta [OS=Homo sapiens]	1
glucose 1,6-bisphosphate synthase [OS=Homo sapiens]	1
putative RNA-binding protein Luc7-like 1 [OS=Homo sapiens]	1
putative tyrosine-protein phosphatase auxilin [OS=Homo sapiens]	1
glia maturation factor gamma [OS=Homo sapiens]	1
Pyridoxal phosphate phosphatase [OS=Homo sapiens]	1
Ras-related protein Rab-35 [OS=Homo sapiens]	1
Rab GTPase-activating protein 1 [OS=Homo sapiens]	1
Enhancer of rudimentary homolog [OS=Homo sapiens]	1
Rab11 family-interacting protein 5 [OS=Homo sapiens]	1

galectin-7 [OS=Homo sapiens]	1
Rab-like protein 3 [OS=Homo sapiens]	1
Galectin-3 [OS=Homo sapiens]	1
fumarylacetoacetate hydrolase domain-containing protein 2A [OS=Homo sapiens]	1
Ran-specific GTPase-activating protein [OS=Homo sapiens]	1
ras-related GTP-binding protein C [OS=Homo sapiens]	1
Gap junction alpha-1 protein [OS=Homo sapiens]	1
S-adenosylhomocysteine hydrolase-like protein 1 [OS=Homo sapiens]	1
Disco-interacting protein 2 homolog B [OS=Homo sapiens]	1
Diphosphoinositol polyphosphate phosphohydrolase 1 [OS=Homo sapiens]	1
Dipeptidyl peptidase 1 [OS=Homo sapiens]	1
Dihydropyrimidinase-related protein 5 [OS=Homo sapiens]	1
dihydropyrimidinase-related protein 4 [OS=Homo sapiens]	1
RING finger protein 32 [OS=Homo sapiens]	1
RNA-binding protein FUS [OS=Homo sapiens]	1
Repulsive guidance molecule A [OS=Homo sapiens]	1
dihydropyrimidinase [OS=Homo sapiens]	1
Double-stranded RNA-specific editase 1 [OS=Homo sapiens]	1
Secernin-1 [OS=Homo sapiens]	1
Secretoglobin family 1D member 1 [OS=Homo sapiens]	1

Secretogranin-2 [OS=Homo sapiens]	1
Secretogranin-3 [OS=Homo sapiens]	1
Secretory carrier-associated membrane protein 1 [OS=Homo sapiens]	1
Dermokine [OS=Homo sapiens]	1
Deoxynucleotidyltransferase terminal-interacting protein 2 [OS=Homo sapiens]	1
rRNA-processing protein UTP23 homolog [OS=Homo sapiens]	1
Electrogenic sodium bicarbonate cotransporter 1 [OS=Homo sapiens]	1
Glutamine synthetase [OS=Homo sapiens]	1
Reticulocalbin-2 [OS=Homo sapiens]	1
Retinal dehydrogenase 1 [OS=Homo sapiens]	1
Retroviral-like aspartic protease 1 [OS=Homo sapiens]	1
elongation factor Tu, mitochondrial [OS=Homo sapiens]	1
rho GDP-dissociation inhibitor 1 [OS=Homo sapiens]	1
Rho GTPase-activating protein 35 [OS=Homo sapiens]	1
ribose-phosphate pyrophosphokinase 1 [OS=Homo sapiens]	1
Rho-related GTP-binding protein RhoC [OS=Homo sapiens]	1
DNA damage-binding protein 1 [OS=Homo sapiens]	1
EF-hand domain-containing protein D1 [OS=Homo sapiens]	1
E3 ubiquitin-protein ligase RBX1 [OS=Homo sapiens]	1
Dynein light chain 2, cytoplasmic [OS=Homo sapiens]	1
Ribonuclease pancreatic [OS=Homo sapiens]	1

Ribonucleoside-diphosphate reductase large subunit [OS=Homo sapiens]	1
Dynactin subunit 5 [OS=Homo sapiens]	1
dual specificity protein phosphatase 3 [OS=Homo sapiens]	1
ER degradation-enhancing alpha-mannosidase-like protein 3 [OS=Homo sapiens]	1
Rho-associated protein kinase 2 [OS=Homo sapiens]	1
Hippocalcin-like protein 4 [OS=Homo sapiens]	1
Histone-lysine N-methyltransferase NSD2 [OS=Homo sapiens]	1
histone H3.3 [OS=Homo sapiens]	1
Protein NipSnap homolog 1 [OS=Homo sapiens]	1
Histone H1.5 [OS=Homo sapiens]	1
Histone H1.0 [OS=Homo sapiens]	1
Histidine--tRNA ligase, cytoplasmic [OS=Homo sapiens]	1
Protein NYNRIN [OS=Homo sapiens]	1
Protein piccolo [OS=Homo sapiens]	1
protein O-GlcNAcase [OS=Homo sapiens]	1
Protein MENT [OS=Homo sapiens]	1
High mobility group protein HMG-I/HMG-Y [OS=Homo sapiens]	1
Protein phosphatase 1 regulatory subunit 7 [OS=Homo sapiens]	1
Protein phosphatase 1A [OS=Homo sapiens]	1
Protein phosphatase methylesterase 1 [OS=Homo sapiens]	1

heterogeneous nuclear ribonucleoproteins A2/B1 [OS=Homo sapiens]	1
Heterogeneous nuclear ribonucleoprotein U [OS=Homo sapiens]	1
Glutamate decarboxylase 1 [OS=Homo sapiens]	1
Histidine triad nucleotide-binding protein 1 [OS=Homo sapiens]	1
Protein FAM171B [OS=Homo sapiens]	1
Immunoglobulin heavy variable 1-69 [OS=Homo sapiens]	1
Immunoglobulin heavy variable 1-45 [OS=Homo sapiens]	1
Protein BRICK1 [OS=Homo sapiens]	1
Protein DDI1 homolog 2 [OS=Homo sapiens]	1
Protein disulfide-isomerase [OS=Homo sapiens]	1
Protein disulfide-isomerase A4 [OS=Homo sapiens]	1
Protein disulfide-isomerase A6 [OS=Homo sapiens]	1
Histone-lysine N-methyltransferase NSD3 [OS=Homo sapiens]	1
Protein FAM166A [OS=Homo sapiens]	1
HLA class I histocompatibility antigen, B-41 alpha chain [OS=Homo sapiens]	1
IgGFc-binding protein [OS=Homo sapiens]	1
Hypoxia up-regulated protein 1 [OS=Homo sapiens]	1
protein FAM49B [OS=Homo sapiens]	1
hydroxymethylglutaryl-CoA synthase, cytoplasmic [OS=Homo sapiens]	1
protein lin-7 homolog B [OS=Homo sapiens]	1

Hyaluronan and proteoglycan link protein 1 [OS=Homo sapiens]	1
Huntingtin-interacting protein K [OS=Homo sapiens]	1
Heterogeneous nuclear ribonucleoprotein H [OS=Homo sapiens]	1
Immunoglobulin epsilon heavy chain [OS=Homo sapiens]	1
Putative annexin A2-like protein [OS=Homo sapiens]	1
G-protein coupled receptor family C group 5 member B [OS=Homo sapiens]	1
Protein-glutamine gamma-glutamyltransferase 5 [OS=Homo sapiens]	1
Glypican-4 [OS=Homo sapiens]	1
Proteoglycan 4 [OS=Homo sapiens]	1
glycolipid transfer protein [OS=Homo sapiens]	1
Glycogen synthase kinase-3 alpha [OS=Homo sapiens]	1
Protocadherin-1 [OS=Homo sapiens]	1
heterogeneous nuclear ribonucleoprotein r [OS=Homo sapiens]	1
Glycerol-3-phosphate dehydrogenase [NAD(+)], cytoplasmic [OS=Homo sapiens]	1
guanine deaminase [OS=Homo sapiens]	1
Glutathione S-transferase P [OS=Homo sapiens]	1
Putative macrophage stimulating 1-like protein [OS=Homo sapiens]	1
glutathione S-transferase mu 4 [OS=Homo sapiens]	1
glutathione S-transferase Mu 3 [OS=Homo sapiens]	1
Putative nucleotidyltransferase FAM46B [OS=Homo sapiens]	1

glutathione S-transferase A5 [OS=Homo sapiens]	1
density-regulated protein [OS=Homo sapiens]	1
purine nucleoside phosphorylase [OS=Homo sapiens]	1
protein Shroom3 [OS=Homo sapiens]	1
heterogeneous nuclear ribonucleoprotein D0 [OS=Homo sapiens]	1
Heterogeneous nuclear ribonucleoprotein A/B [OS=Homo sapiens]	1
Protein S100-A13 [OS=Homo sapiens]	1
Heparan-sulfate 6-O-sulfotransferase 3 [OS=Homo sapiens]	1
Protein S100-A16 [OS=Homo sapiens]	1
Heme-binding protein 1 [OS=Homo sapiens]	1
Protein S100-P [OS=Homo sapiens]	1
GRIP1-associated protein 1 [OS=Homo sapiens]	1
Protein Shroom1 [OS=Homo sapiens]	1
GTP:AMP phosphotransferase AK3, mitochondrial [OS=Homo sapiens]	1
Heat shock 70 kDa protein 12A [OS=Homo sapiens]	1
Protein TFG [OS=Homo sapiens]	1
Guanine nucleotide-binding protein-like 1 [OS=Homo sapiens]	1
Guanine nucleotide-binding protein subunit alpha-12 [OS=Homo sapiens]	1
Protein Z-dependent protease inhibitor [OS=Homo sapiens]	1
protein/nucleic acid deglycase DJ-1 [OS=Homo sapiens]	1

Guanine nucleotide-binding protein G(I)/G(S)/G(O) subunit gamma-2 [OS=Homo sapiens]	1
Glutaminase kidney isoform, mitochondrial [OS=Homo sapiens]	1
Protein SET [OS=Homo sapiens]	1

Supplementary Table 2.

David gene functional annotation (UP_Tissue)

Tissue	count	%	P-value	Benjamini
Liver	321	62.3	2.30E-63	2.90E-61
Cajal-Retzius cell	74	14.4	2.80E-63	2.90E-61
Fetal brain cortex	76	14.8	1.10E-62	7.40E-61
Brain	330	64.1	2.90E-22	1.50E-20
Erythroleukemia	218	42.3	1.40E-21	5.80E-20
Lymphoblast	46	8.9	6.10E-18	2.10E-16
Cervix carcinoma	225	43.7	2.00E-16	5.80E-15
Platelet	52	10.1	4.60E-15	1.20E-13
Colon carcinoma	47	9.1	3.60E-11	8.20E-10
Lung	124	24.1	1.00E-09	2.10E-08
Muscle	55	10.7	2.10E-09	4.00E-08

B-cell lymphoma	19	3.7	6.30E-08	1.10E-06
Cerebellum	60	11.7	8.20E-08	1.20E-06
Leukemic T-cell	110	21.4	8.30E-08	1.20E-06
Uterus	85	16.5	1.70E-06	2.40E-05
Amygdala	47	9.1	7.70E-06	9.90E-05
Mammary carcinoma	14	2.7	8.90E-06	1.10E-04
Lymph	39	7.6	1.70E-05	1.90E-04
Placenta	136	26.4	3.20E-05	3.50E-04
Embryonic kidney	34	6.6	3.50E-05	3.70E-04
Ovarian carcinoma	17	3.3	7.10E-05	7.00E-04
Hippocampus	39	7.6	1.20E-04	1.20E-03
Skin	76	14.8	1.30E-04	1.20E-03
Adipocyte	7	1.4	2.60E-04	2.20E-03
Fetal brain	42	8.2	3.00E-04	2.50E-03
Eye	48	9.3	4.30E-04	3.50E-03
Amygdala	24	4.7	4.60E-04	3.50E-03
Fetal whole brain	10	1.9	4.70E-04	3.50E-03
Skeletal muscle	32	6.2	1.30E-03	9.20E-03
T-cell	19	3.7	1.30E-03	9.20E-03
Brain cortex	13	2.5	2.70E-03	1.80E-02
Pituitary	11	2.1	4.60E-03	2.90E-02
